# Supplementary material for: LARP1 binds ribosomes and TOP mRNAs in repressed complexes
Source: EMBO J. 2024 Nov 12;43(24):6555–72. doi: 10.1038/s44318-024-00294-z (PMC11649897; doi:10.1038/s44318-024-00294-z)
Supplement: Supplementary file 1 — Appendix [file 44318_2024_294_MOESM1_ESM.pdf]

Appendix for

LARP1 binds ribosomes and TOP mRNAs in repressed complexes

Authors: James A. Saba[1,2,†], Zixuan Huang[3,†], Kate L. Schole[1,2], Xianwen Ye[3], Shrey D. Bhatt[1,2], Yi Li[3], Winston Timp[1,4], Jingdong Cheng[3*], Rachel Green[1,2*]

Affiliations:

[1] Department of Molecular Biology and Genetics, Johns Hopkins University School of Medicine, Baltimore, MD 21205, USA.

[2] Howard Hughes Medical Institute, Johns Hopkins University School of Medicine, Baltimore, MD 21205, USA.

[3] Minhang Hospital & Institutes of Biomedical Sciences, Shanghai Key Laboratory of Medical Epigenetics, International Co-laboratory of Medical Epigenetics and Metabolism, Fudan University, Dong'an Road 131, 200032, Shanghai, China.

[4] Department of Biomedical Engineering, Johns Hopkins University, Baltimore, MD 21218, USA.

[†] These authors contributed equally.

*Corresponding authors. Email: cheng@fudan.edu.cn (J.C.), ragreen@jhmi.edu (R.G.)

Table of Contents

1. Appendix Text (page 3)
2. Appendix Figures S1 to S10 (pages 4-23)
3. Appendix Table S1 (page 24-25)
4. Appendix References (page 26)

Appendix Text

TOP-80S shift in high-K^+ gradients

Previous studies have shown that vacant 40S and 60S subunit couples are held together more weakly than ribosomes actively engaged with mRNAs, peptidyl-tRNAs or initiation complexes (Infante & Baierlein, 1971; Beller & Lubsen, 1972; Noll *et al*, 1973). As a result, these “vacant couples” are more susceptible to splitting by high hydrostatic pressure as they travel through sucrose gradients. Hydrostatic pressure follows the following formula:

$$P = \rho g h$$

where P = hydrostatic pressure, ρ = fluid density, g = acceleration due to gravity, and h = fluid depth. From this formula it becomes clear that as depth in the gradient increases, the hydrostatic pressure proportionally increases. In high-K^+ gradients, the high potassium concentration further weakens the general intersubunit interactions of vacant couples. As a result, they initially sediment as an 80S ribosome. Once they travel deep enough in the gradient such that the hydrostatic pressure overcomes the force of intersubunit attraction between the 40S and 60S subunits, they split and then begin sedimenting more slowly as independent 40S and 60S subunits. Ultimately this combination of initial migration as an 80S followed by migration as individual subunits causes them and the associated TOP mRNA to sediment in between a normal 40S and 80S distribution.

LARP1 isoforms: While the 1019 amino acid isoform of LARP1 (ENSEMBL LARP1-201) has been referenced extensively in past literature (Philippe *et al*, 2018; Fonseca *et al*, 2015; Kozlov *et al*, 2022; Jia *et al*, 2021; Lahr *et al*, 2017; Hong *et al*, 2017), the 1096 amino acid isoform (ENSEMBL LARP1-204) appears to be the major isoform expressed in most cell types (Schwenzer *et al*, 2021). Importantly, the two constructs differ only at their disordered N-termini (amino acids 1-144 of the LARP1-204 construct), are identical at characterized domains including the La/PAM2, RBR, and DM15 domains, and are thought to be functionally equivalent. We used the 1019 amino acid isoform (LARP1-201) for expression of WT-, RBRmut-, and LPDmut constructs. However, all of our annotations and numbering throughout the manuscript correspond to the 1096 amino acid isoform (LARP1-204) to maintain consistency with the isoform that appears to be majorly expressed in human cells (Schwenzer *et al*, 2021).

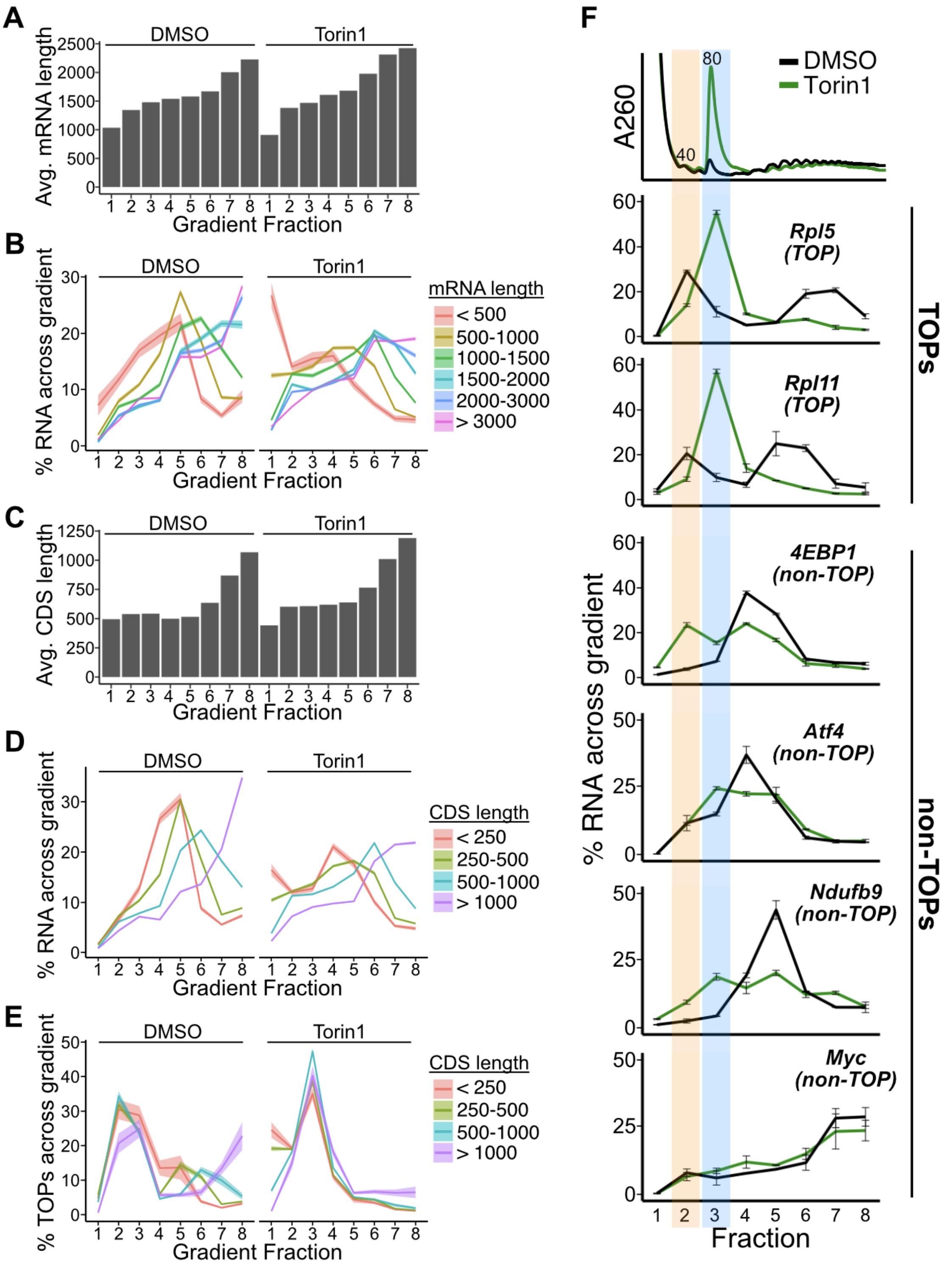
A
DMSO
Torin1
Avg. mRNA length
Gradient Fraction
B
mRNA length
< 500
500-1000
1000-1500
1500-2000
2000-3000
> 3000
% RNA across gradient
C
Avg. CDS length
D
CDS length
< 250
250-500
500-1000
> 1000
E
% TOPs across gradient
F
A260
40
80
DMSO
Torin1
Rpl5 (TOP)
Rpl11 (TOP)
TOPs
4EBP1 (non-TOP)
Atf4 (non-TOP)
Ndufb9 (non-TOP)
Myc (non-TOP)
non-TOPs
Fraction

Appendix Figure S1. Analysis and validation of nanopore sequencing data.
(**A**) Average mRNA length from each sample of the nanopore sequencing data. Each read was weighted according to the annotated transcript length (ENSEMBL). (**B**) Average distribution across the gradient for mRNAs, binned by their annotated transcript length. Values are centered at the average +/- SEM (lighter shade). (**C**) Same as (A) except that reads were weighted according to their annotated CDS length. (**D**) Same as (B) except that reads were binned by their annotated CDS length. (**E**) Same as (D), except for TOPs only. (**F**) qPCR for additional TOPs and non-TOPs from Fig 1C. For clarity, the A260 trace is reproduced from Fig 1C. Error bars are centered at the average and represent the SD from 2-4 technical replicates. For (A-E), only reads aligning to the ENSEMBL canonical transcript were used in the analysis. Annotated transcript and CDS lengths are for the ENSEMBL canonical transcript.

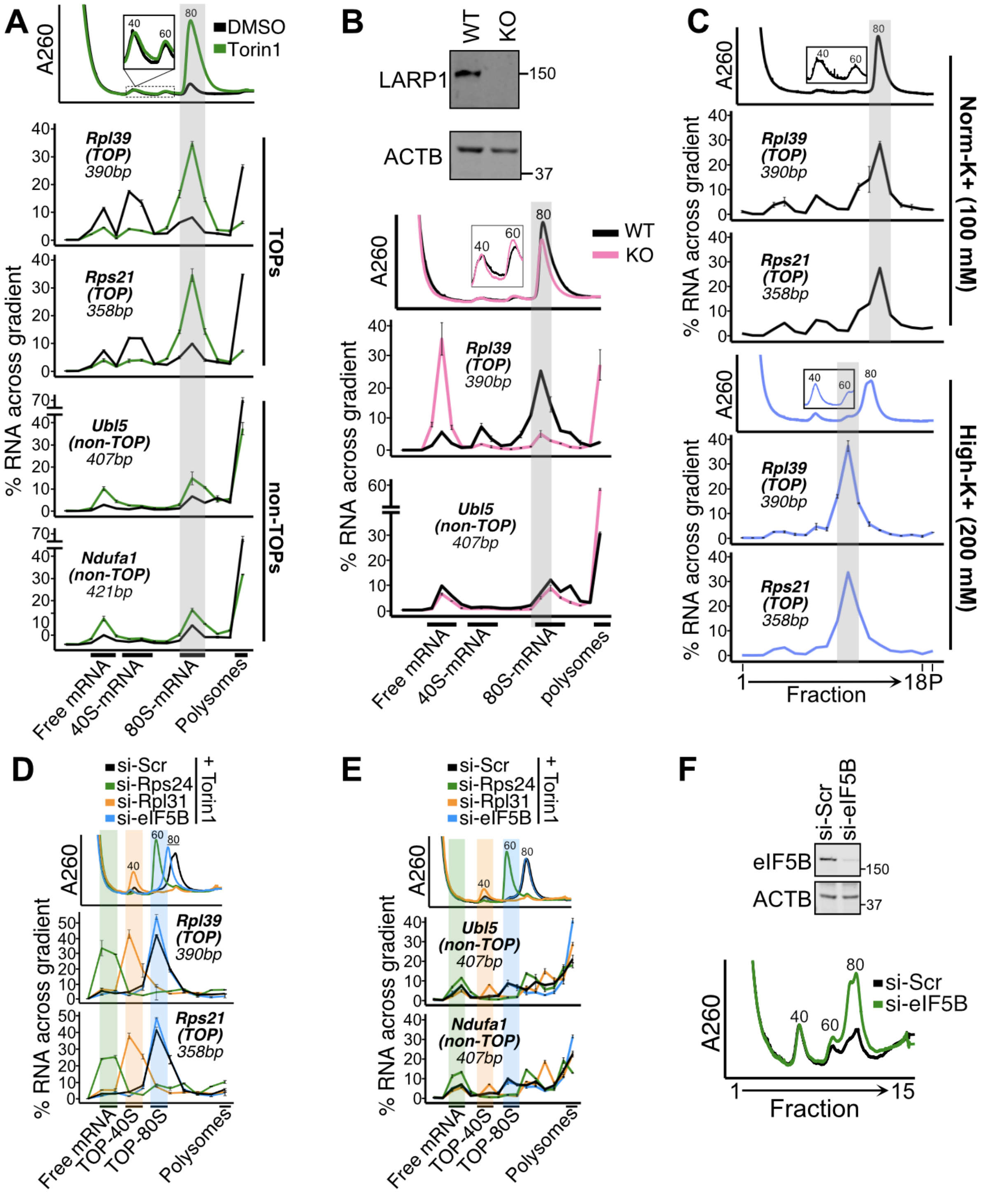
A
B
C
D
E
F
DMSO
Torin1
A260
% RNA across gradient
Rpl39 (TOP) 390bp
Rps21 (TOP) 358bp
Ubl5 (non-TOP) 407bp
Ndufa1 (non-TOP) 421bp
TOPs
non-TOPs
Free mRNA
40S-mRNA
80S-mRNA
Polysomes
WT
KO
LARP1
ACTB
150
37
Norm-K+ (100 mM)
High-K+ (200 mM)
Fraction
18P
si-Scr
si-Rps24
si-Rpl31
si-eIF5B
+ Torin1
TOP-40S
TOP-80S
eIF5B
15

Appendix Figure S2. Characterization of the TOP-80S complex (related to Figure 2).
(A) Biological replicate experiment for Fig 2A. (**B**) WT and LARP1-KO cells (HEK293T) were treated with 300 nM Torin1 for 1 h and lysates spread along 15-35% sucrose gradients containing 100 mM KOAc followed by qPCR against genes of interest. Western blot validating the LARP1-KO is shown. (**C**) Biological replicate experiment for Fig 2B. **(D)** Biological replicate experiment for Fig 2C. **(E)** qPCR for additional genes from Fig 2C. For clarity, the A260 trace is reproduced from Fig 2C. (**F**) HEK293T cells were treated with siRNAs targeting scrambled (Si-Scr) or eIF5B for 48 hours and lysates fractionated along 15-35% sucrose gradients containing 200 mM KOAc. Equal RNA was loaded for each sample. Western blot demonstrating knockdown efficiency is shown. For (A-C), gray highlights correspond to the TOP-80S. For (D-E), green, orange, and blue highlights correspond to free mRNA, the TOP-40S and the TOP-80S, respectively. For qPCR plots, error bars are centered at the average and represent the SD from 2-4 technical replicates.

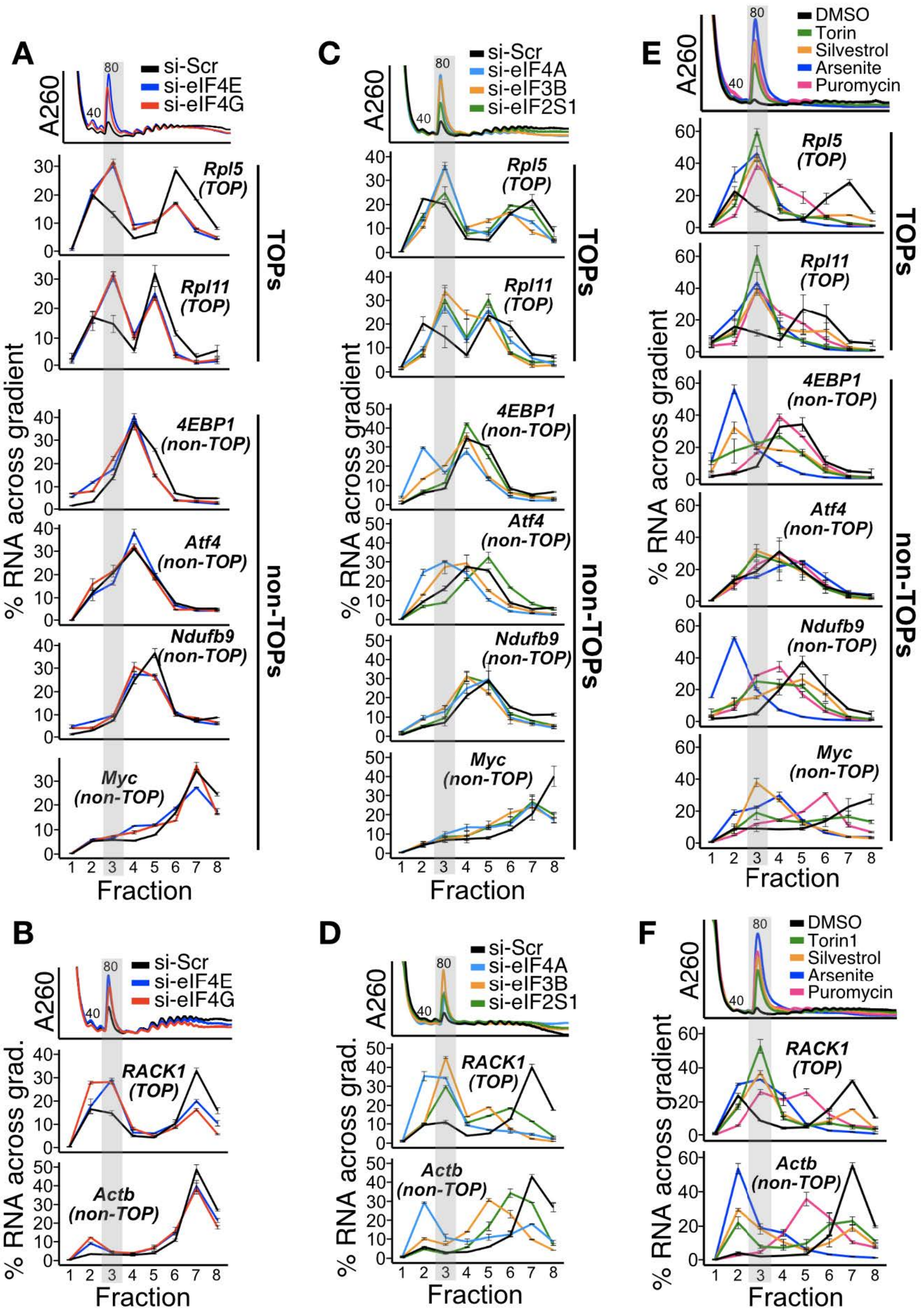
A
si-Scr
si-eIF4E
si-eIF4G
A260
40
80
Rpl5 (TOP)
Rpl11 (TOP)
TOPs
4EBP1 (non-TOP)
Atf4 (non-TOP)
Ndufb9 (non-TOP)
Myc (non-TOP)
non-TOPs
% RNA across gradient
Fraction
B
si-Scr
si-eIF4E
si-eIF4G
RACK1 (TOP)
Actb (non-TOP)
% RNA across grad.
% RNA across gradient
Fraction
C
si-Scr
si-eIF4A
si-eIF3B
si-eIF2S1
Rpl5 (TOP)
Rpl11 (TOP)
TOPs
4EBP1 (non-TOP)
Atf4 (non-TOP)
Ndufb9 (non-TOP)
Myc (non-TOP)
non-TOPs
% RNA across gradient
Fraction
D
si-Scr
si-eIF4A
si-eIF3B
si-eIF2S1
RACK1 (TOP)
Actb (non-TOP)
% RNA across grad.
% RNA across gradient
Fraction
E
DMSO
Torin
Silvestrol
Arsenite
Puromycin
Rpl5 (TOP)
Rpl11 (TOP)
TOPs
4EBP1 (non-TOP)
Atf4 (non-TOP)
Ndufb9 (non-TOP)
Myc (non-TOP)
non-TOPs
% RNA across gradient
Fraction
F
DMSO
Torin1
Silvestrol
Arsenite
Puromycin
RACK1 (TOP)
Actb (non-TOP)
% RNA across gradient
Fraction

Appendix Figure S3. Increases in free ribosomes drive TOP- 80S formation (related to Figure 3).

(**A-B**) qPCR for additional TOPs and non-TOPs (A) and biological replicate experiment (B) for Fig 3A. (**C-D**) qPCR for additional TOPs and non-TOPs (C) and biological replicate experiment (D) for Fig 3B. (**E-F**) qPCR for additional TOPs and non-TOPs (E) and biological replicate experiment (F) for Fig 3C. For (A-F), gray highlights correspond to the TOP-80S. For qPCR plots, error bars are centered at the average and represent the SD from 2-4 technical replicates. For (A, C, and E), A260 traces are reproduced from their respective main figures for clarity.

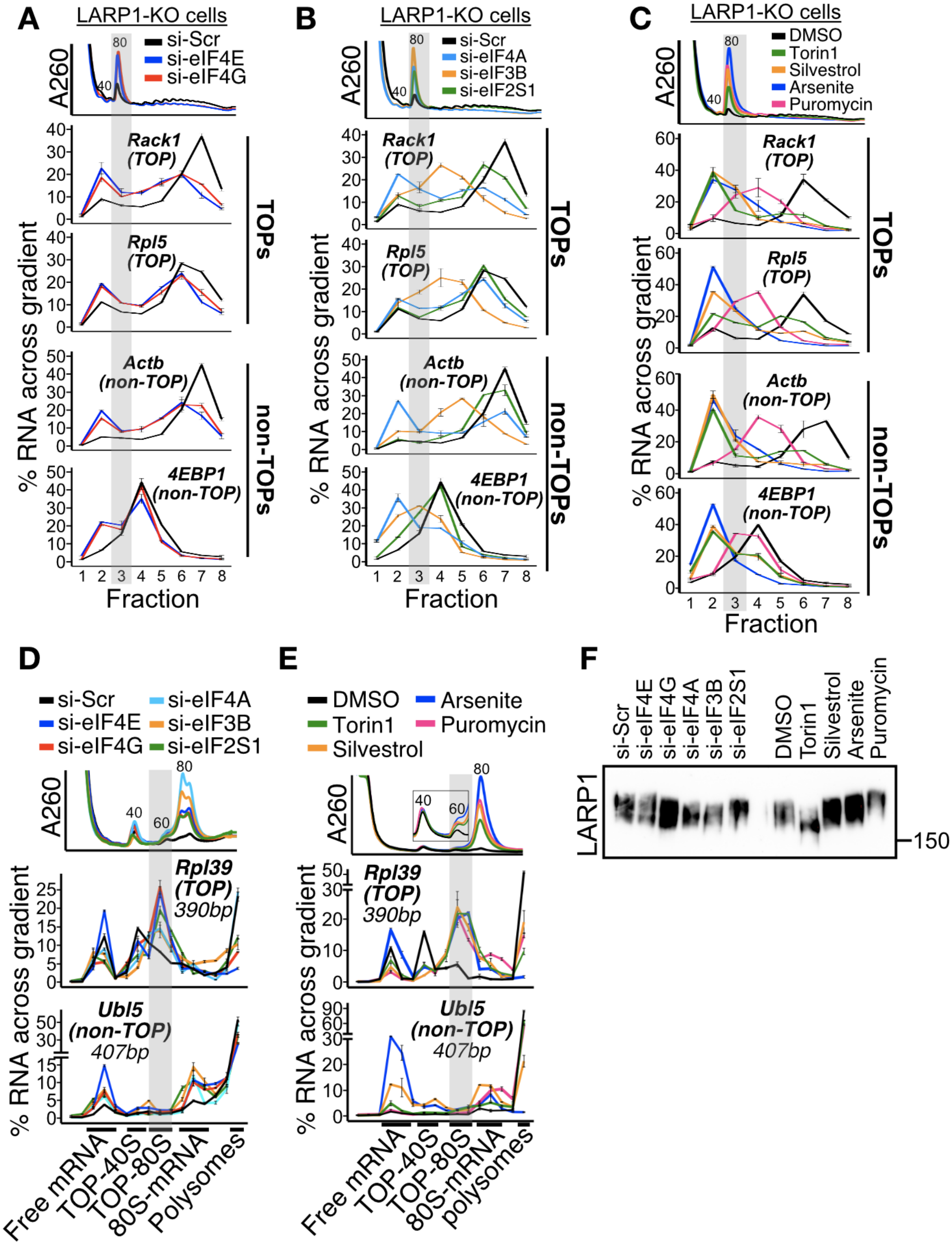
A
LARP1-KO cells
si-Scr
si-eIF4E
si-eIF4G
A260
Rack1 (TOP)
Rpl5 (TOP)
Actb (non-TOP)
4EBP1 (non-TOP)
TOPs
non-TOPs
% RNA across gradient
Fraction
B
LARP1-KO cells
si-Scr
si-eIF4A
si-eIF3B
si-eIF2S1
C
LARP1-KO cells
DMSO
Torin1
Silvestrol
Arsenite
Puromycin
D
si-Scr
si-eIF4E
si-eIF4G
si-eIF4A
si-eIF3B
si-eIF2S1
Rpl39 (TOP) 390bp
Ubl5 (non-TOP) 407bp
Free mRNA
TOP-40S
TOP-80S
80S-mRNA
Polysomes
E
DMSO
Torin1
Silvestrol
Arsenite
Puromycin
polysomes
F
LARP1
150

Appendix Figure S4. TOP-80S complexes are LARP1-dependent and weakly-associated. (**A**) LARP1-KO cells were treated with siRNAs targeting scrambled (si-Scr), eIF4E, or eIF4G and lysates fractionated along 10-50% sucrose gradients followed by qPCR against genes of interest. (**B**) Identical to (A) except with siRNAs targeting scrambled (si-Scr), eIF4A1/2, eIF3B, or eIF2S1. (**C**) Identical to (A) except cells were treated with Torin1 (300 nM, 1 h), Silvestrol (30 nM, 1 h), Sodium arsenite (Arsenite; 100 µM, 1 h), or Puromycin (250 µM, 30 min). (**D**) WT HEK293T cells were treated with siRNAs targeting scrambled (si-Scr), eIF4E, eIF4G, eIF4A1/2, eIF3B, or eIF2S1 and lysates fractionated along 15-35% sucrose gradients containing 200 mM KOAc (High-K^+) followed by qPCR against genes of interest. (**E**) Identical to (C) except cells were treated with Torin1 (300 nM, 1 h), Silvestrol (30 nM, 1 h), Sodium arsenite (Arsenite; 100 µM, 1 h), or Puromycin (250 µM, 30 min). (**F**) Biological replicate experiment for Fig 3D. For (A-E), gray highlights correspond to the TOP-80S fraction. For qPCR plots, error bars are centered at the average and represent the SD from 2-4 technical replicates.

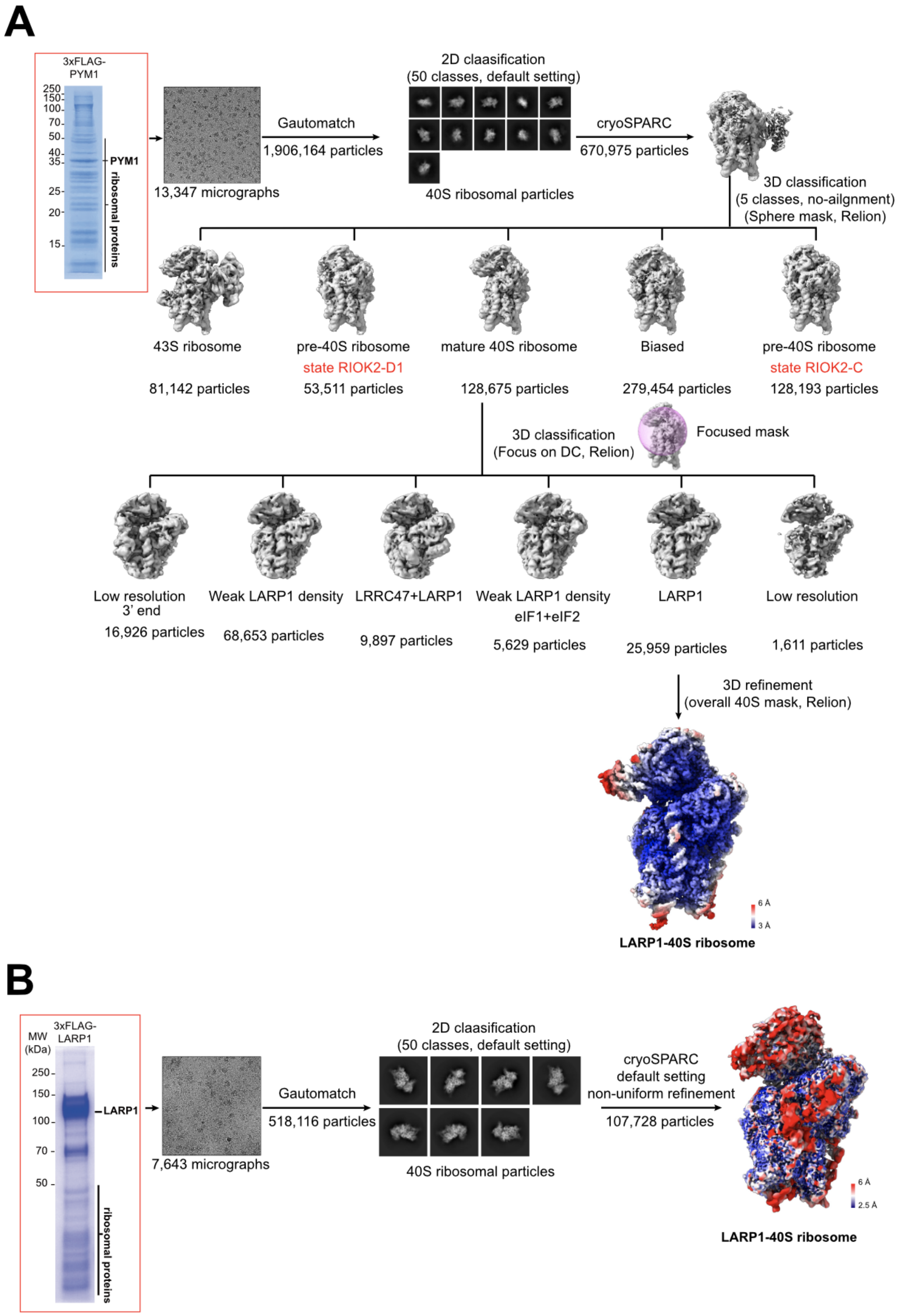
A
3xFLAG-PYM1
250
150
100
70
50
40
35
25
20
15
PYM1
ribosomal proteins
13,347 micrographs
Gautomatch
1,906,164 particles
2D claasification
(50 classes, default setting)
40S ribosomal particles
cryoSPARC
670,975 particles
3D classification
(5 classes, no-ailgnment)
(Sphere mask, Relion)
43S ribosome
81,142 particles
pre-40S ribosome
state RIOK2-D1
53,511 particles
mature 40S ribosome
128,675 particles
Biased
279,454 particles
pre-40S ribosome
state RIOK2-C
128,193 particles
3D classification
(Focus on DC, Relion)
Focused mask
Low resolution
3' end
16,926 particles
Weak LARP1 density
68,653 particles
LRRC47+LARP1
9,897 particles
Weak LARP1 density
eIF1+eIF2
5,629 particles
LARP1
25,959 particles
Low resolution
1,611 particles
3D refinement
(overall 40S mask, Relion)
6 Å
3 Å
LARP1-40S ribosome
B
MW (kDa)
3xFLAG-LARP1
250
150
100
70
50
LARP1
ribosomal proteins
7,643 micrographs
Gautomatch
518,116 particles
2D claasification
(50 classes, default setting)
40S ribosomal particles
cryoSPARC
default setting
non-uniform refinement
107,728 particles
6 Å
2.5 Å
LARP1-40S ribosome

Appendix Figure S5. The sorting scheme of the cryo-EM dataset.
(**A-B**) Sorting schemes of the cryo-EM datasets from the PYM1 (A) and LARP1 (B) immunoprecipitation samples. Masks and software used during data processing are shown. Maps are colored according to their local resolution distribution.

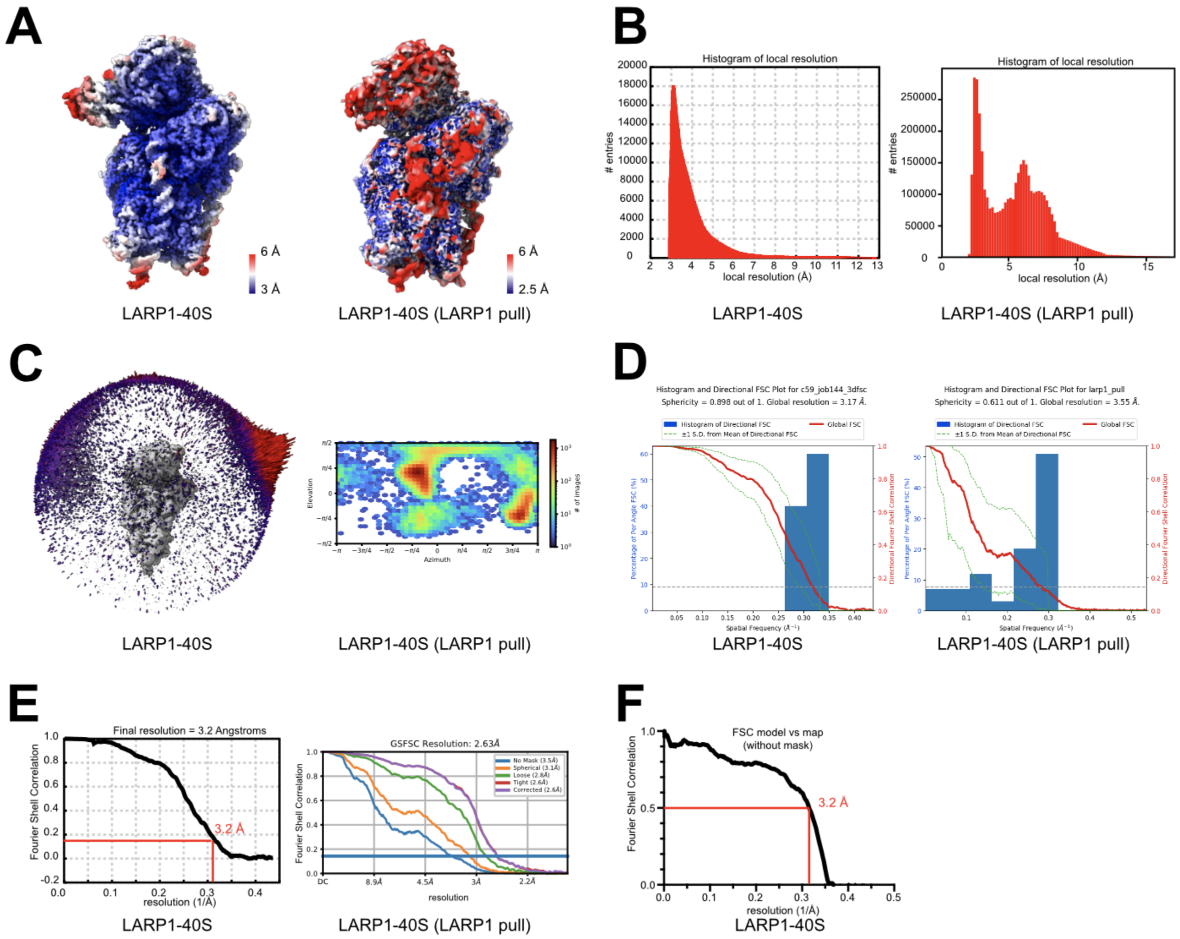
A
6 Å
3 Å
LARP1-40S
6 Å
2.5 Å
LARP1-40S (LARP1 pull)
B
Histogram of local resolution
entries
local resolution (Å)
LARP1-40S
Histogram of local resolution
entries
local resolution (Å)
LARP1-40S (LARP1 pull)
C
Elevation
Azimuth
# of images
LARP1-40S
LARP1-40S (LARP1 pull)
D
Histogram and Directional FSC Plot for c59_job144_3dfsc
Sphericity = 0.898 out of 1. Global resolution = 3.17 Å.
Histogram and Directional FSC Plot for larp1_pull
Sphericity = 0.611 out of 1. Global resolution = 3.55 Å.
LARP1-40S
LARP1-40S (LARP1 pull)
E
Final resolution = 3.2 Angstroms
Fourier Shell Correlation
resolution (1/Å)
3.2 Å
LARP1-40S
GSFSC Resolution: 2.63Å
No Mask (3.5Å)
Spherical (3.1Å)
Loose (2.6Å)
Tight (2.6Å)
Corrected (2.6Å)
Fourier Shell Correlation
resolution
LARP1-40S (LARP1 pull)
F
FSC model vs map
(without mask)
Fourier Shell Correlation
resolution (1/Å)
3.2 Å
LARP1-40S

Appendix Figure S6. Structural analysis and validation of the LARP1-40S structures.
(**A**) Cryo-EM maps of the LARP1-40S ribosomes from the PYM1 (left) and LARP1 (right) samples, colored according to their local resolution estimation. (**B-C**) Local resolution distribution plots (B) and angular distributions of the particles (C) used for the final LARP1-40S (left) and LARP1-40S (LARP1 pull, right) reconstructions. The LARP1-40S plots were generated using Relion, while the LARP1-40S (LARP1 pull) plots were generated using cryoSPARC. (**D**) Corresponding 3D Fourier Shell Correlation (FSC) curves calculated in 3DFSC. (**E-F**) Standard FSC curves of the maps (E) and model-to-map correlation curves (F) calculated in Relion. Resolutions were estimated using either the 0.143 or 0.5 cutoff criterion (red dotted line).

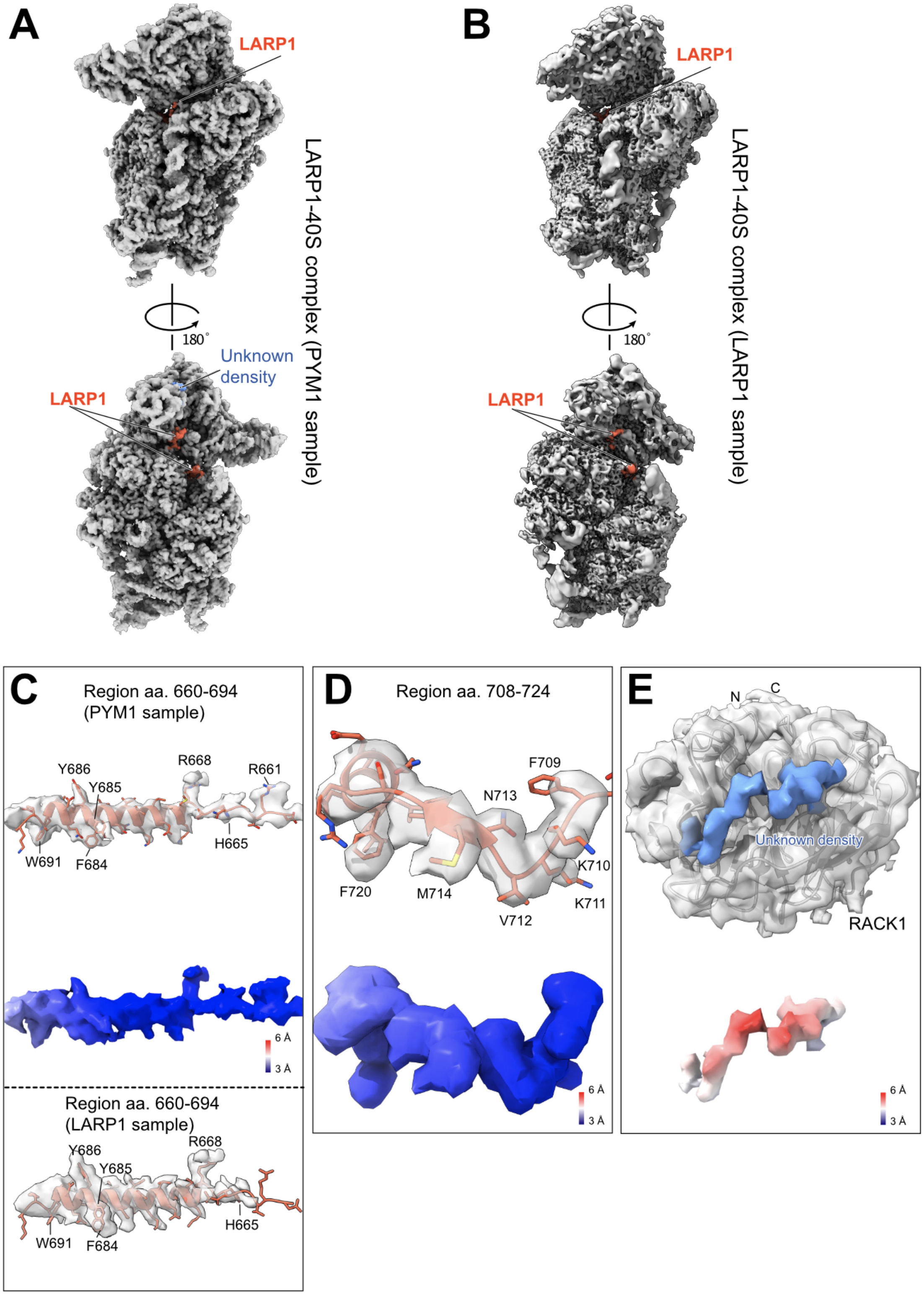

A
LARP1
LARP1-40S complex (PYM1 sample)
180°
Unknown density
LARP1
B
LARP1
LARP1-40S complex (LARP1 sample)
180°
LARP1
C
Region aa. 660-694
(PYM1 sample)
Y686
Y685
R668
R661
H665
W691
F684
6 Å
3 Å
Region aa. 660-694
(LARP1 sample)
Y686
Y685
R668
H665
W691
F684
D
Region aa. 708-724
F709
N713
K710
K711
V712
M714
F720
6 Å
3 Å
E
N
C
Unknown density
RACK1
6 Å
3 Å

Appendix Figure S7. Detailed analysis of the interaction between LARP1 and the 40S ribosome.

(**A-B**) Two different views of the density maps of the LARP1-40S ribosome structures obtained from the PYM1 sample (A) and the LARP1 sample (B). The maps are either filtered by DeepEMhancer (A) or cryoSPARC (B) according to their local resolution estimation. LARP1 (tomato red) is indicated. (**C-E**) Molecular models for LARP1 residues 660-694 (C), 708-724 (D), and the unknown density on RACK1 (E) are shown with density maps to support the assignment. The density map for region 660-694 is derived from the unsharpened map. Their relative maps are colored according to the local resolution estimated by Relion. Region 660-694 from the structure obtained from the LARP1 sample is also shown (C, bottom)

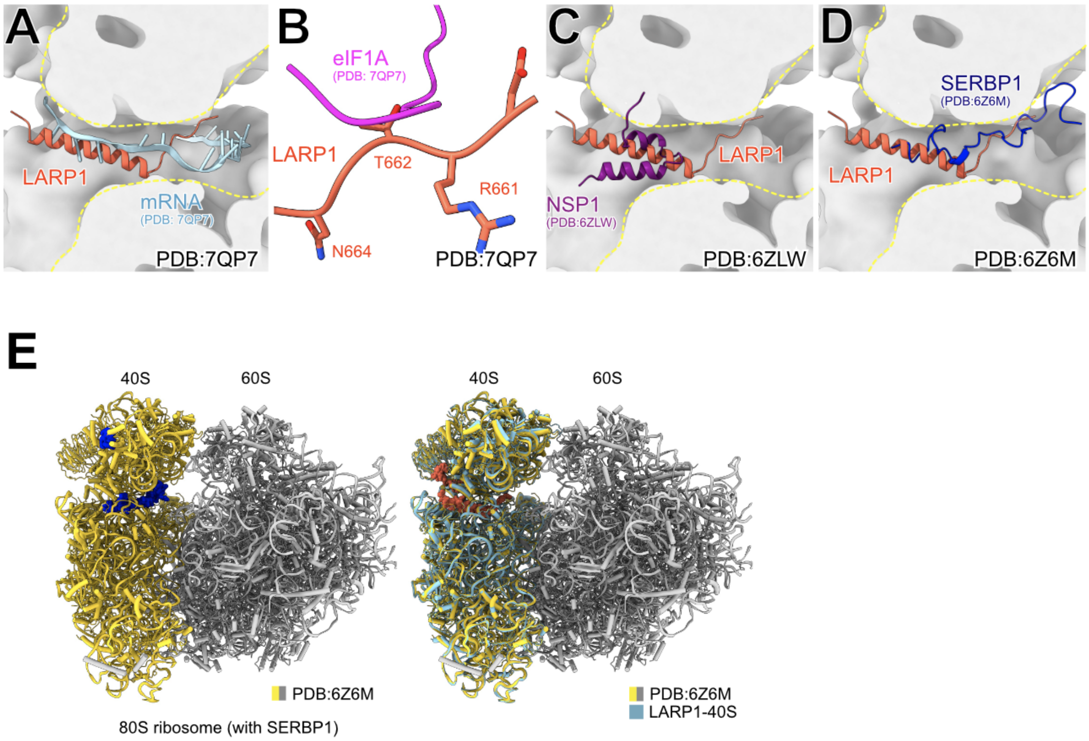
A
LARP1
mRNA
(PDB: 7QP7)
PDB:7QP7
B
eIF1A
(PDB: 7QP7)
LARP1
T662
R661
N664
PDB:7QP7
C
LARP1
NSP1
(PDB:6ZLW)
PDB:6ZLW
D
SERBP1
(PDB:6Z6M)
LARP1
PDB:6Z6M
E
40S
60S
PDB:6Z6M
80S ribosome (with SERBP1)
40S
60S
PDB:6Z6M
LARP1-40S

Appendix Figure S8. The LARP1 RBR occludes mRNA binding.
(**A-B**) Structural comparison of the LARP1-40S ribosome with the 48S initiation complex (PDB: 7QP7). The LARP1 RBR spatially clashes with mRNA in the mRNA channel (A) and with eIF1A near the decoding center (B). (**C-D**) Structural comparison of the LARP1 RBR with NSP1 (C) and SERBP1 (D) when bound in the mRNA channel. (**E**) Structural comparison of SERBP1-80S and LARP1-40S structures. Left: overall structure of the SERBP1-80S complex (PDB: 6Z6M). SERBP1 is colored in blue, while the 40S and 60S subunits are colored in yellow and gray, respectively. eEF2 and E-tRNA are not shown. Right: superimposition of the LARP1-40S structure (40S: light blue) with the SERBP1-80S structure (40S: yellow; 60S: gray), indicating that LARP1 does not spatially impede 80S formation. LARP1 is colored in tomato red.

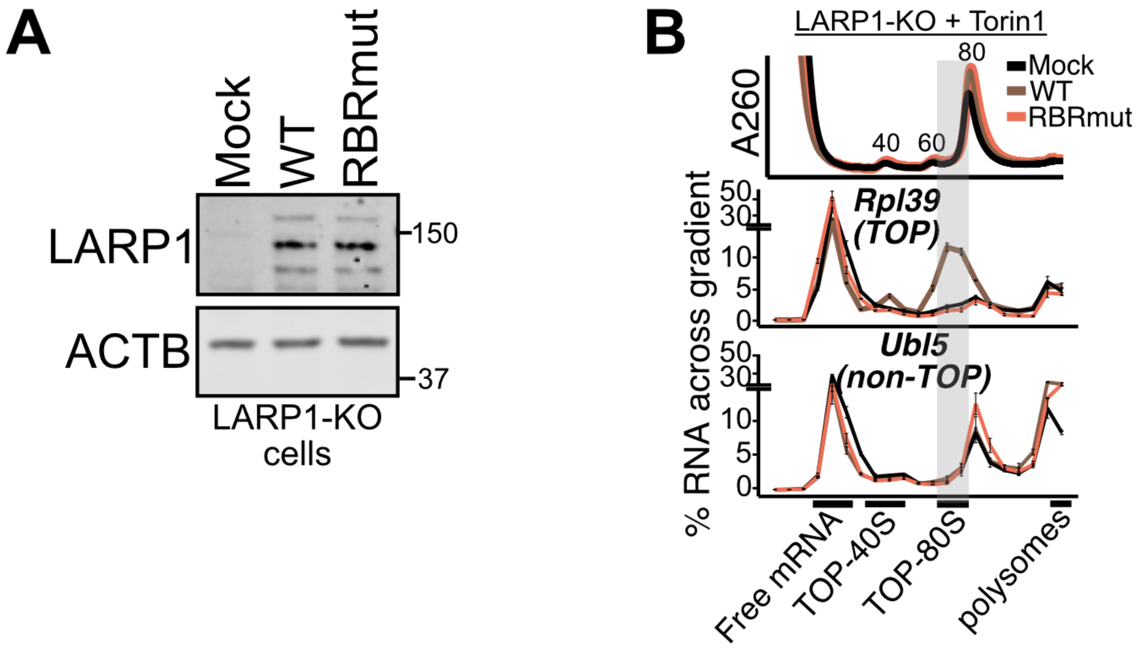
A
Mock
WT
RBRmut
LARP1
150
ACTB
37
LARP1-KO cells
B
LARP1-KO + Torin1
A260
40
60
80
Mock
WT
RBRmut
% RNA across gradient
Rpl39 (TOP)
Ubl5 (non-TOP)
50
30
10
5
0
Free mRNA
TOP-40S
TOP-80S
polysomes

Appendix Figure S9. RBRmut-LARP1 does not form the TOP-80S.
(**A**) Biological replicate experiment for Fig 5B showing equal protein expression of WT- and RBRmut-LARP1 constructs expressed in LARP1-KO cells. (**B**) Biological replicate experiment for Fig 5C showing RBRmut-LARP1 is incapable of forming the TOP-40S or TOP-80S complexes. Gray highlights correspond to the TOP-80S fraction. Error bars are centered at the average and represent the SD from 2-4 technical replicates.

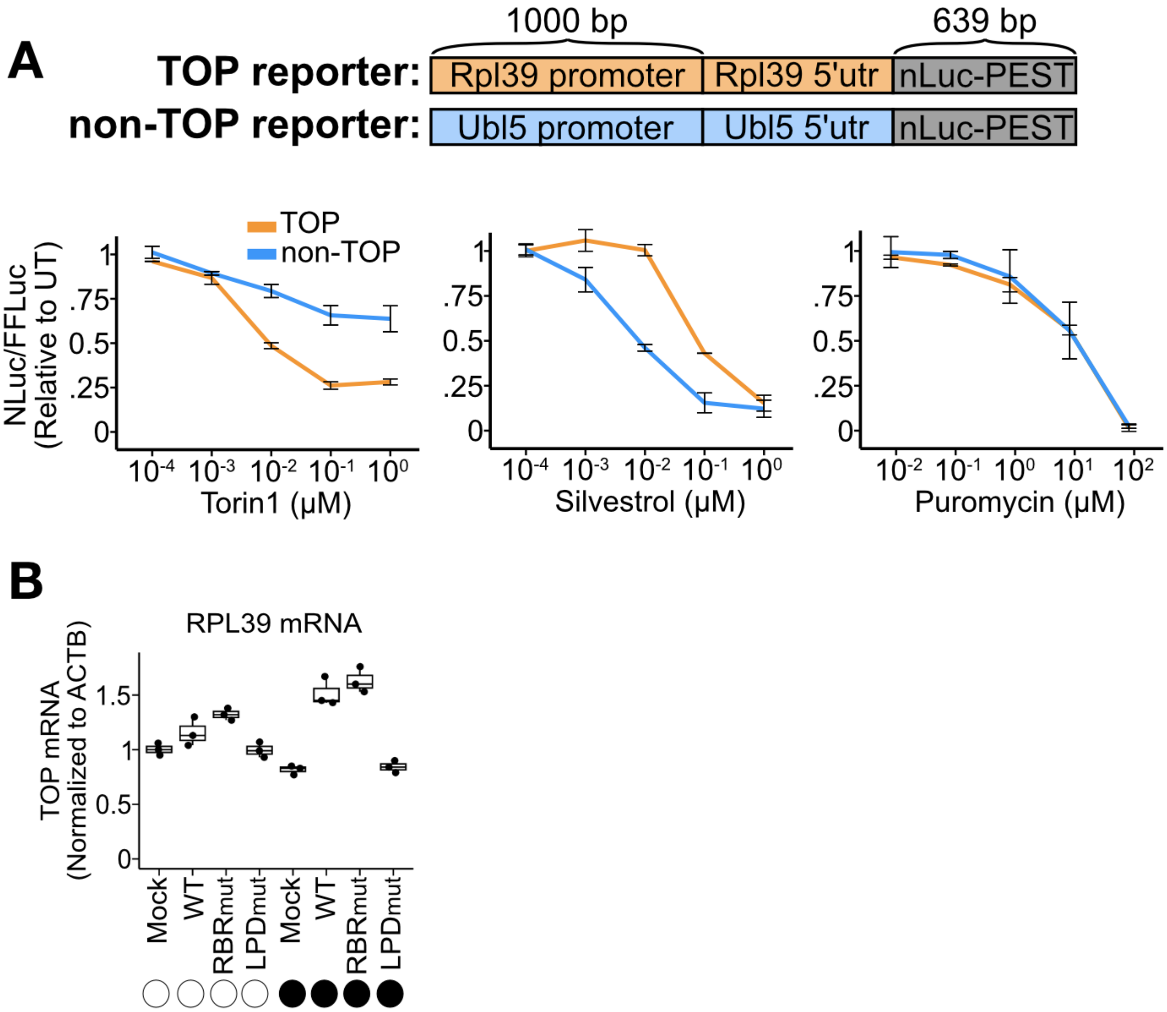
A
1000 bp
639 bp
TOP reporter:
Rpl39 promoter
Rpl39 5'utr
nLuc-PEST
non-TOP reporter:
Ubl5 promoter
Ubl5 5'utr
nLuc-PEST
TOP
non-TOP
NLuc/FFLuc (Relative to UT)
Torin1 (µM)
Silvestrol (µM)
Puromycin (µM)
B
RPL39 mRNA
TOP mRNA (Normalized to ACTB)
Mock
WT
RBRmut
LPDmut
Mock
WT
RBRmut
LPDmut

Appendix Figure S10. Free ribosomes do not drive TOP repression or stabilization.
(**A**) Top: Schematic (reproduced from Fig 6A) showing reporter constructs. FFluc expressed from a CMV promoter on the same plasmid served as a transfection control. Bottom: NLuc-PEST expression (normalized to FFLuc) from HEK293T cells treated with the indicated treatments at the indicated concentrations for 90 minutes. Values were normalized to untreated. Error bars are centered at the average and represent the SD of three biological replicate experiments (**B**) Steady state mRNA levels (qPCR) from three biological replicates for RPL39 normalized to ACTB mRNA. Values were normalized to the mock-transfected, untreated sample. Torin1 treatment was 300 nM for 24 hours. For box plots: whiskers represent minima and maxima; bounds of box represent the quartiles (25th and 75th percentile); center line represents the median.

Appendix Table S1. Cryo-EM data collection, refinement, and validation statistics.

	LARP1+40S ribosome (PDB 8XP2) (EMDB 38548)	LARP1+40S ribosome (LARP1 pull) (EMDB 38550)
**Data collection and Processing (for each dataset)**		
Microscope	Titan Krios G4	Titan Krios G4
Voltage (keV)	300	300
Camera	Falcon IV	Falcon IV
Magnification	105,000	130,000
Pixel size at detector (Å/pixel)	1.146	0.932
Total electron exposure ($e^{-}/Å^2$)	~ 50	~58
Exposure rate (e-/pixel/sec)	~ 12	~12
Number of frames collected during exposure	1737 (EER format)	1080 (EER format)
Defocus range (μm)	-1 to -2.5	-1 to -2.5
Phase plate (if used)	N/A	N/A
- phase shift range (in degrees)		
- number of images per phase plate position		
Automation software (EPU, SerialEM or manual)	EPU 2	EPU 2
Tilt angle (if grid was tilted)	N/A	N/A
Energy filter slit width (if used)	20 eV	20 eV
Micrographs collected (no.)	15,497	7,768
Micrographs used (no.)	13,347	7,643
Total extracted particles (no.)	1,906,164	518,116
**For each reconstruction:**		
Refined particles (no.)	25,959	107,728
Final particles (no.)	25,959	107,728
Point-group or helical symmetry parameters	*C1*	*C1*
Estimated error of translations/rotations (if available)	N/A	N/A
Resolution (global, Å)	9.3/3.9	8.2/3.0
FSC 0.5 (unmasked/masked)	4.7/3.2	3.5/2.6
FSC 0.143 (unmasked/masked)	2.9-13	2.5-15
Resolution range (local, Å)	2.9-3.6	2.2-13
Resolution range due to anisotropy (Å)	-66	N/A
Map sharpening *B* factor ($Å^2$)	Local resolution	N/A
Map sharpening methods	filter	
**Model composition (for each model)**		N/A
Protein	4,957	
Ligands	2	
RNA	1,740	
**Model Refinement (for each model)**		
Refinement package	Phenix	

- real or reciprocal space	Real
- resolution cutoff	3.2
Model-Map scores	
-CC	0.78
- Average FSC	0.70
B factors (Å^2)	72.98
Protein residues	71.54
Ligands	120.53
RNA	74.52
R.m.s. deviations from ideal values	
Bond lengths (Å)	0.003
Bond angles (°)	0.786
**Validation (for each model)**	
MolProbity score	1.63
CaBLAM outliers	2.92
Clashscore	5.87
Poor rotamers (%)	0.05
C-beta deviations (%)	0.02
EMRinger score (if better than 4 Å resolution)	2.39
Ramachandran plot	
Favored (%)	95.61
Outliers (%)	0.10

Appendix References

Beller RJ & Lubsen NH (1972) Effect of polypeptide chain length on dissociation of ribosomal complexes. *Biochemistry* 11: 3271–3276

Fonseca BD, Zakaria C, Jia J-J, Graber TE, Svitkin Y, Tahmasebi S, Healy D, Hoang H-D, Jensen JM, Diao IT, *et al* (2015) La-related Protein 1 (LARP1) Represses Terminal Oligopyrimidine (TOP) mRNA Translation Downstream of mTOR Complex 1 (mTORC1). *J Biol Chem* 290: 15996–16020

Hong S, Freeberg MA, Han T, Kamath A, Yao Y, Fukuda T, Suzuki T, Kim JK & Inoki K (2017) LARP1 functions as a molecular switch for mTORC1-mediated translation of an essential class of mRNAs. *Elife* 6

Infante AA & Baierlein R (1971) Pressure-induced dissociation of sedimenting ribosomes: effect on sedimentation patterns. *Proc Natl Acad Sci U S A* 68: 1780–1785

Jia J-J, Lahr RM, Solgaard MT, Moraes BJ, Pointet R, Yang A-D, Celucci G, Graber TE, Hoang H-D, Niklaus MR, *et al* (2021) mTORC1 promotes TOP mRNA translation through site-specific phosphorylation of LARP1. *Nucleic Acids Res* 49: 3461–3489

Kozlov G, Mattijssen S, Jiang J, Nyandwi S, Sprules T, Iben JR, Coon SL, Gaidamakov S, Noronha AM, Wilds CJ, *et al* (2022) Structural basis of 3'-end poly(A) RNA recognition by LARP1. *Nucleic Acids Res* 50: 9534–9547

Lahr RM, Fonseca BD, Ciotti GE, Al-Ashtal HA, Jia J-J, Niklaus MR, Blagden SP, Alain T & Berman AJ (2017) La-related protein 1 (LARP1) binds the mRNA cap, blocking eIF4F assembly on TOP mRNAs. *Elife* 6: e24146

Noll M, Hapke B, Schreier MH & Noll H (1973) Structural dynamics of bacterial ribosomes. I. Characterization of vacant couples and their relation to complexed ribosomes. *J Mol Biol* 75: 281–294

Philippe L, Vasseur J-J, Debart F & Thoreen CC (2018) La-related protein 1 (LARP1) repression of TOP mRNA translation is mediated through its cap-binding domain and controlled by an adjacent regulatory region. *Nucleic Acids Res* 46: 1457–1469

Schwenzer H, Abdel Mouti M, Neubert P, Morris J, Stockton J, Bonham S, Fellermeyer M, Chettle J, Fischer R, Beggs AD, *et al* (2021) LARP1 isoform expression in human cancer cell lines. *RNA Biol* 18: 237–247
