# Supplementary figures and images for: LARP1 binds ribosomes and TOP mRNAs in repressed complexes
Source: EMBO J. 2024 Nov 12;43(24):6555–72. doi: 10.1038/s44318-024-00294-z (PMC11649897; doi:10.1038/s44318-024-00294-z)

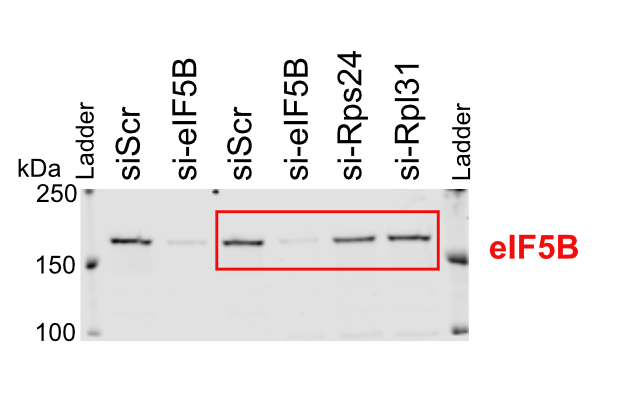

Supplement: Supplementary file 4 — Source data Fig. 2 [file 44318_2024_294_MOESM4_ESM.zip › Figure_2/2C/western_eIF5B.tiff]

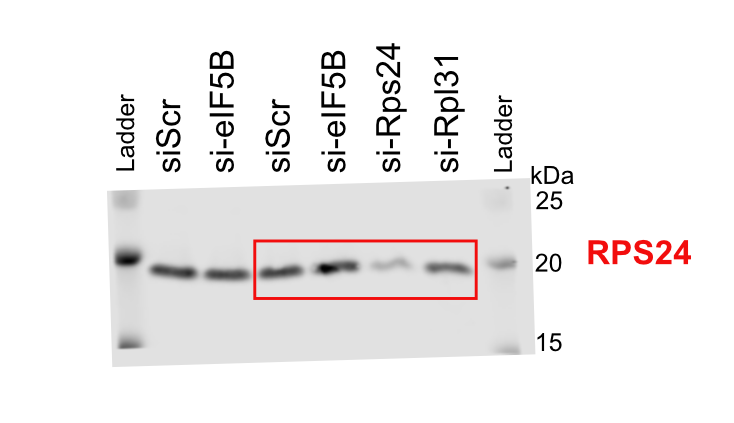

Supplement: Supplementary file 4 — Source data Fig. 2 [file 44318_2024_294_MOESM4_ESM.zip › Figure_2/2C/western_RPS24.tiff]

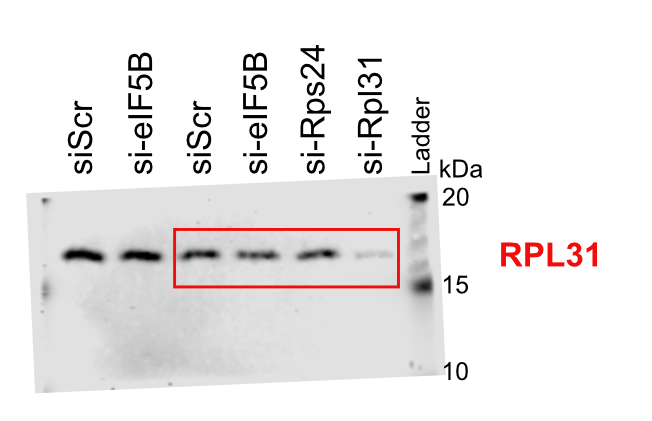

Supplement: Supplementary file 4 — Source data Fig. 2 [file 44318_2024_294_MOESM4_ESM.zip › Figure_2/2C/western_RPL31.tiff]

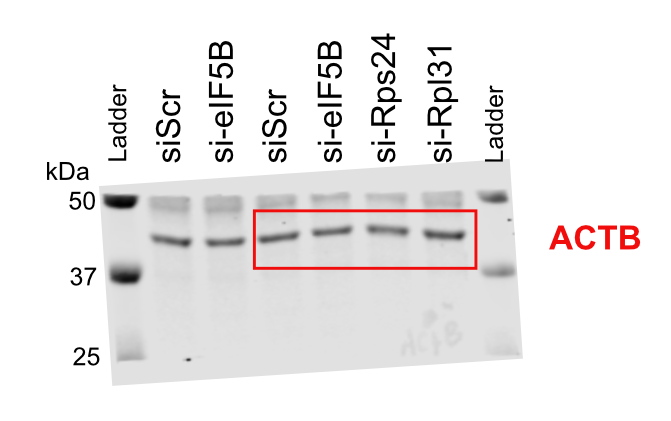

Supplement: Supplementary file 4 — Source data Fig. 2 [file 44318_2024_294_MOESM4_ESM.zip › Figure_2/2C/western_ACTB.tiff]

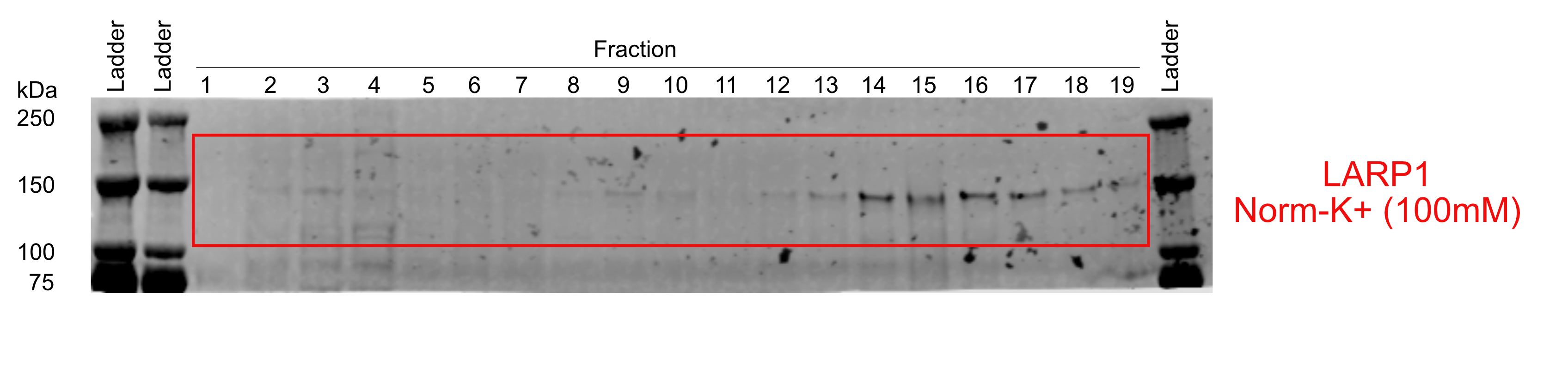

Supplement: Supplementary file 4 — Source data Fig. 2 [file 44318_2024_294_MOESM4_ESM.zip › Figure_2/2B/western_LARP1_100mM.tiff]

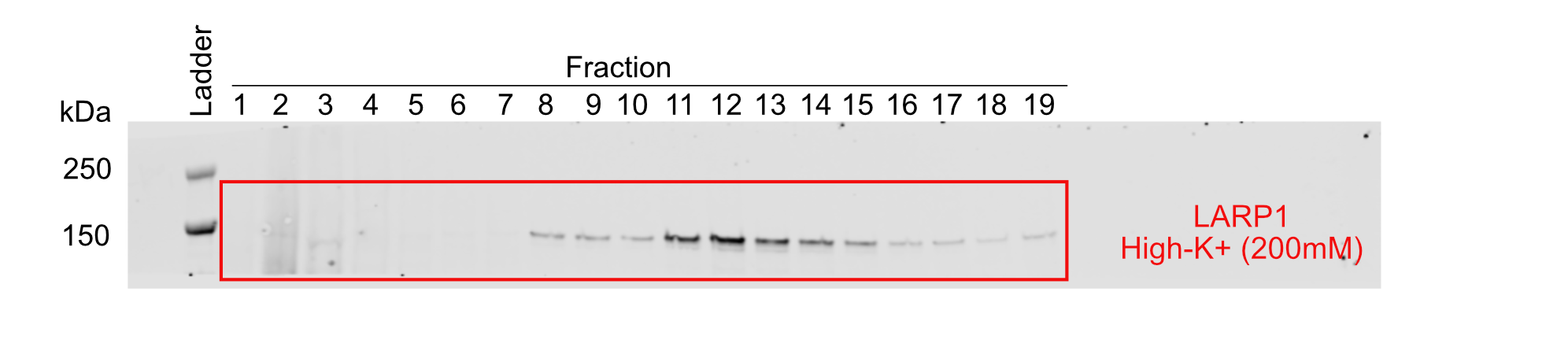

Supplement: Supplementary file 4 — Source data Fig. 2 [file 44318_2024_294_MOESM4_ESM.zip › Figure_2/2B/western_LARP1_200mM.tiff]

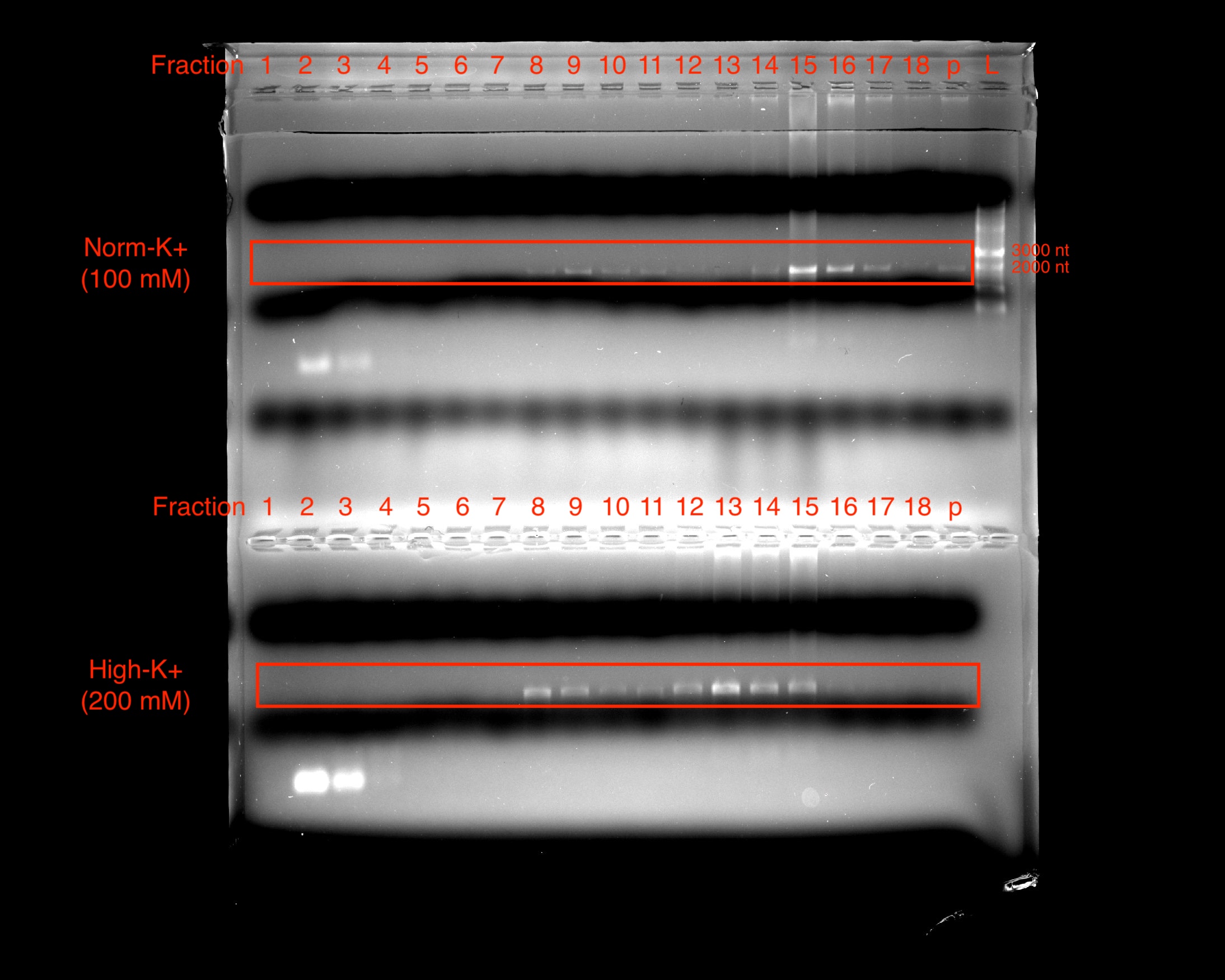

Supplement: Supplementary file 4 — Source data Fig. 2 [file 44318_2024_294_MOESM4_ESM.zip › Figure_2/2B/18S-EtBr-gel_100mM-200mM.jpg]

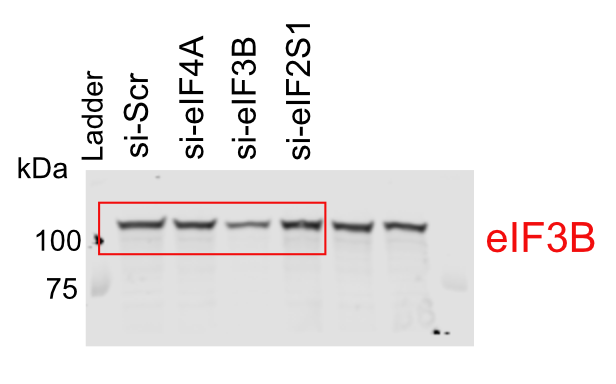

Supplement: Supplementary file 5 — Source data Fig. 3 [file 44318_2024_294_MOESM5_ESM.zip › Figure_3/3B/western_eIF3B.tiff]

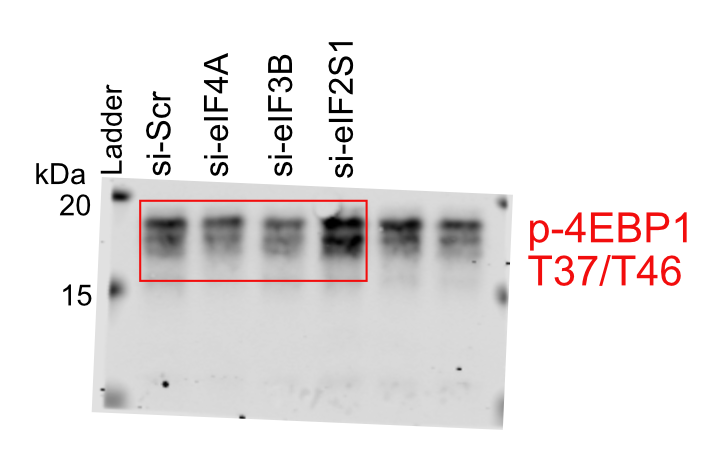

Supplement: Supplementary file 5 — Source data Fig. 3 [file 44318_2024_294_MOESM5_ESM.zip › Figure_3/3B/western_p-4EBP1-t37-t46.tiff]

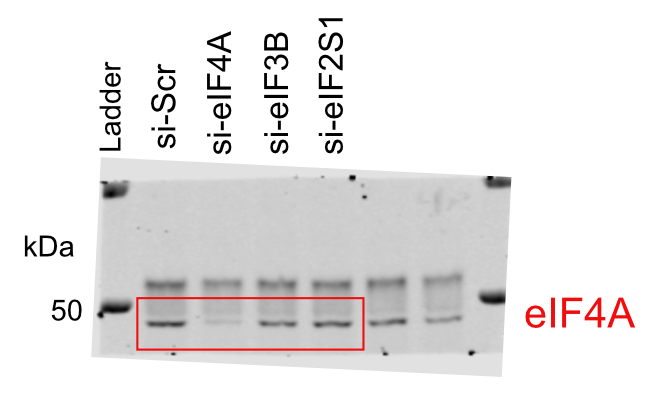

Supplement: Supplementary file 5 — Source data Fig. 3 [file 44318_2024_294_MOESM5_ESM.zip › Figure_3/3B/western_eIF4A.tiff]

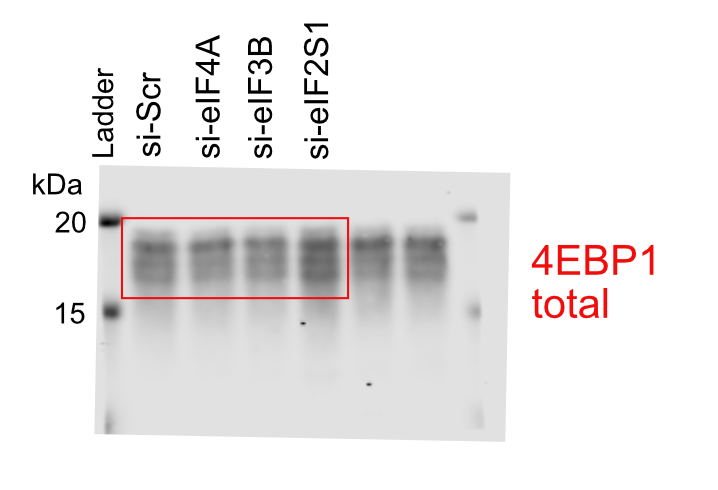

Supplement: Supplementary file 5 — Source data Fig. 3 [file 44318_2024_294_MOESM5_ESM.zip › Figure_3/3B/western_4EBP1-total.tiff]

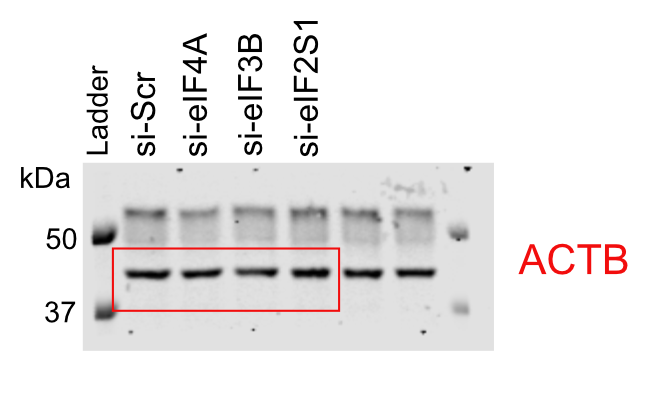

Supplement: Supplementary file 5 — Source data Fig. 3 [file 44318_2024_294_MOESM5_ESM.zip › Figure_3/3B/western_ACTB.tiff]

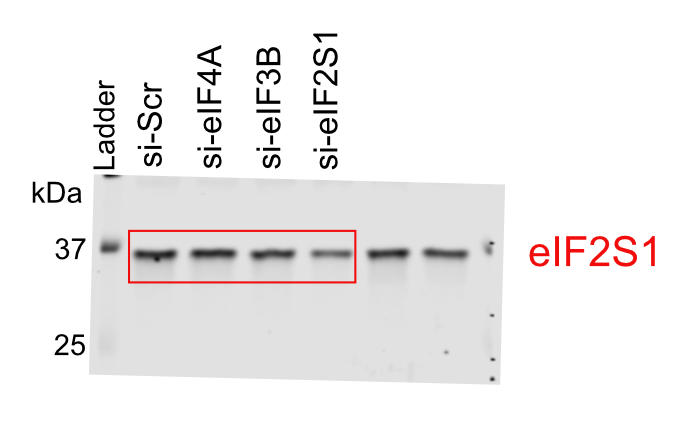

Supplement: Supplementary file 5 — Source data Fig. 3 [file 44318_2024_294_MOESM5_ESM.zip › Figure_3/3B/western_eIF2S1.tiff]

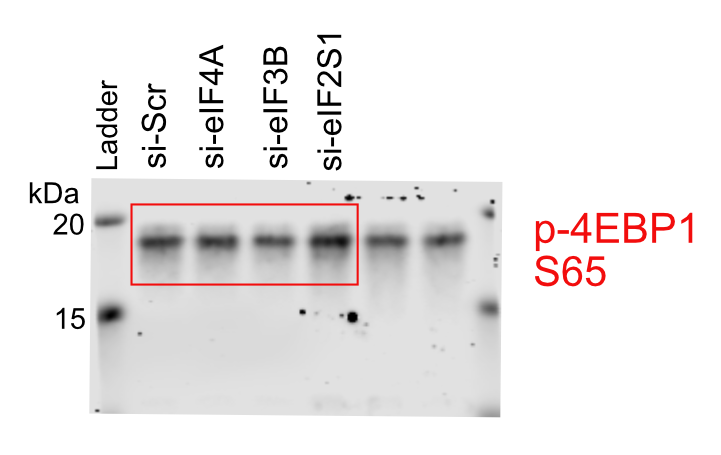

Supplement: Supplementary file 5 — Source data Fig. 3 [file 44318_2024_294_MOESM5_ESM.zip › Figure_3/3B/western_p-4EBP1-s65.tiff]

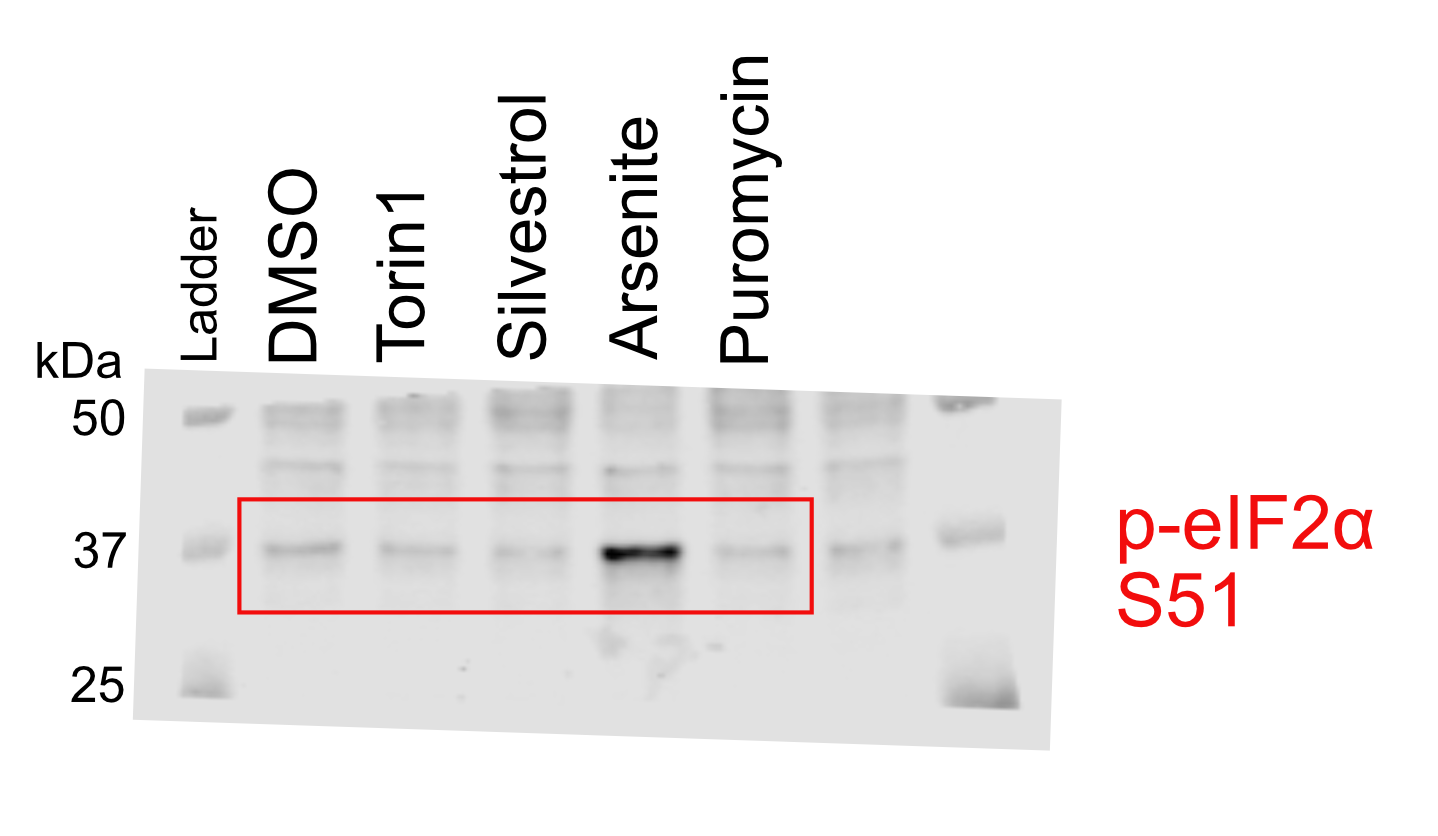

Supplement: Supplementary file 5 — Source data Fig. 3 [file 44318_2024_294_MOESM5_ESM.zip › Figure_3/3C/western_p-eIF2a-S51.tiff]

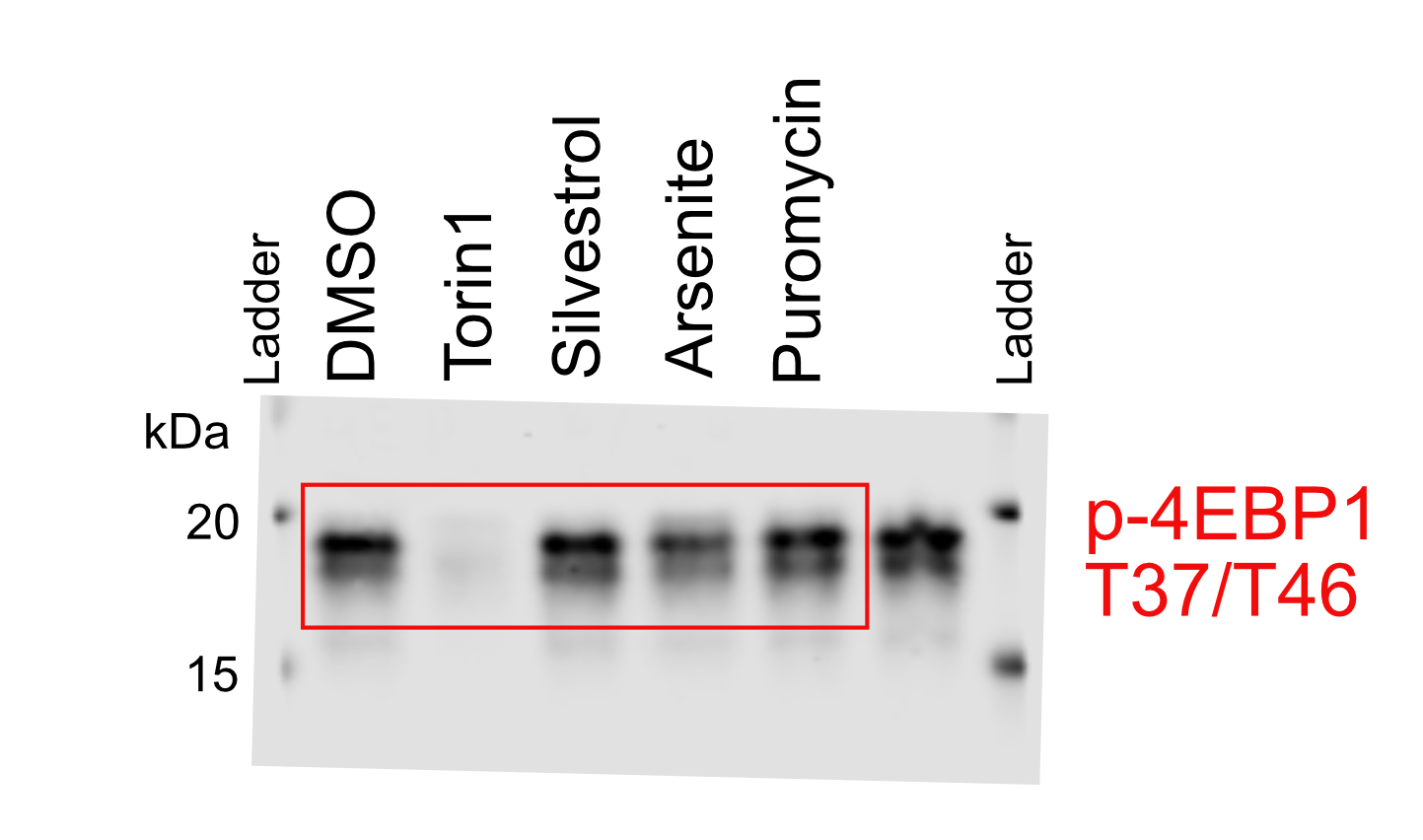

Supplement: Supplementary file 5 — Source data Fig. 3 [file 44318_2024_294_MOESM5_ESM.zip › Figure_3/3C/western_p-4EBP1-t37-t46.tiff]

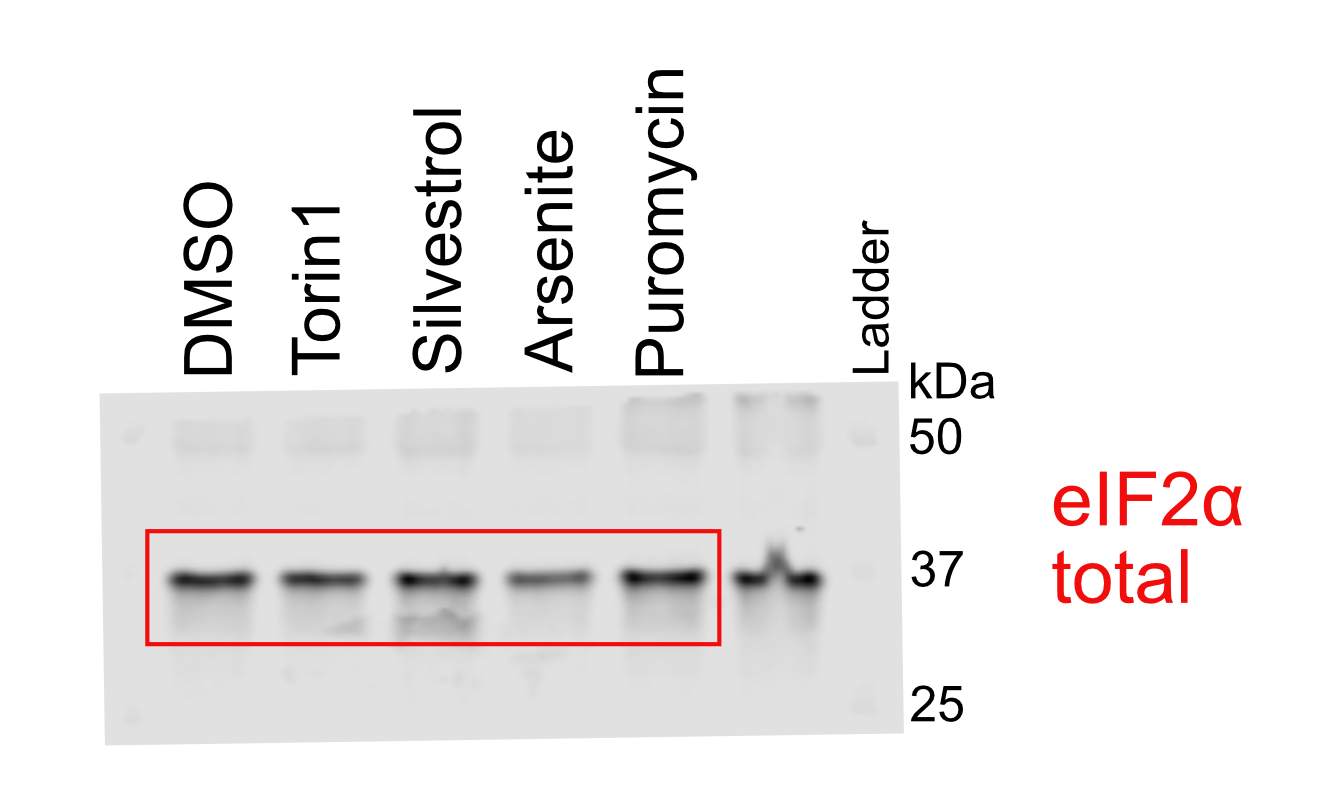

Supplement: Supplementary file 5 — Source data Fig. 3 [file 44318_2024_294_MOESM5_ESM.zip › Figure_3/3C/western_eIF2a-total.tiff]

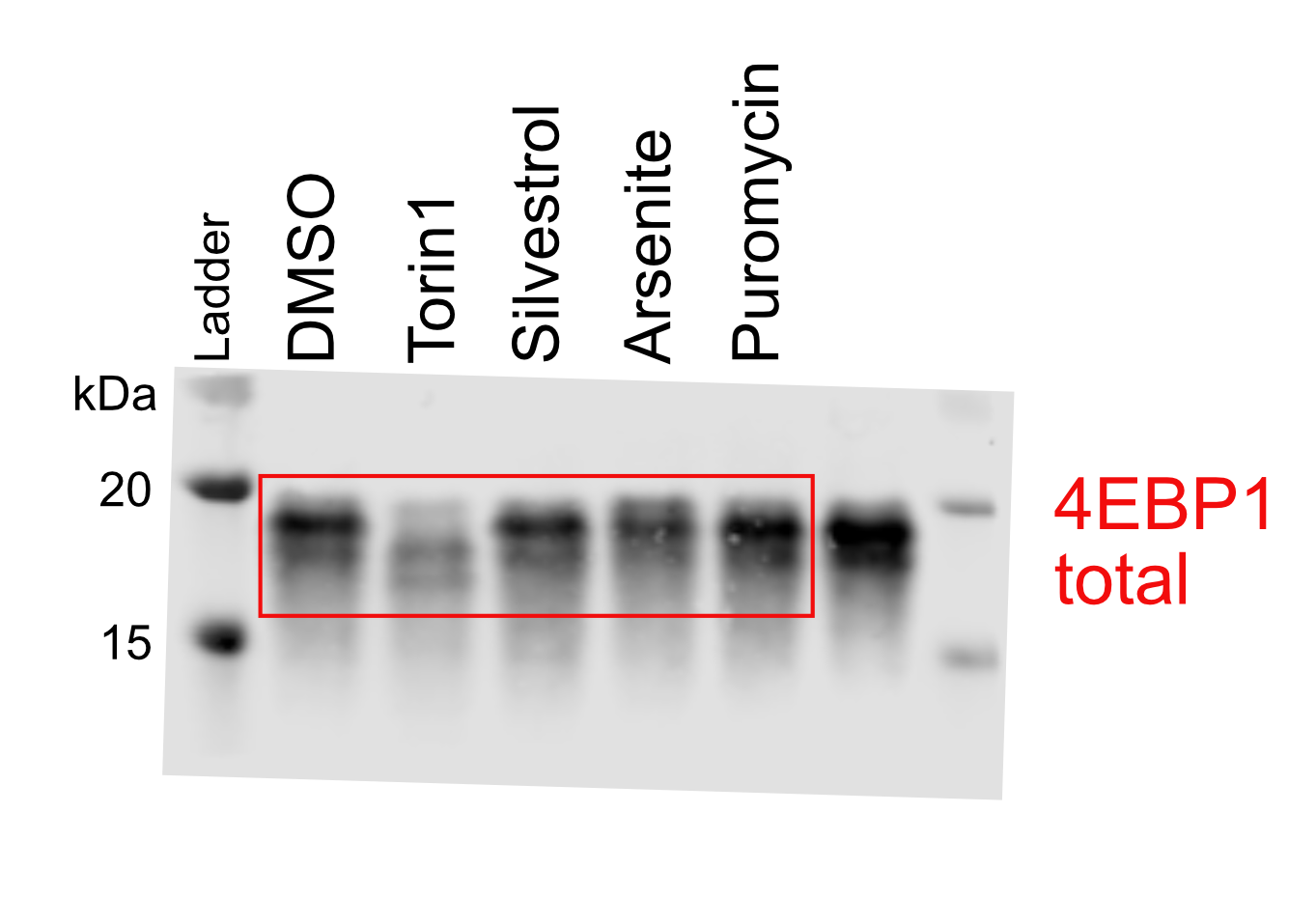

Supplement: Supplementary file 5 — Source data Fig. 3 [file 44318_2024_294_MOESM5_ESM.zip › Figure_3/3C/western_4EBP1-total.tiff]

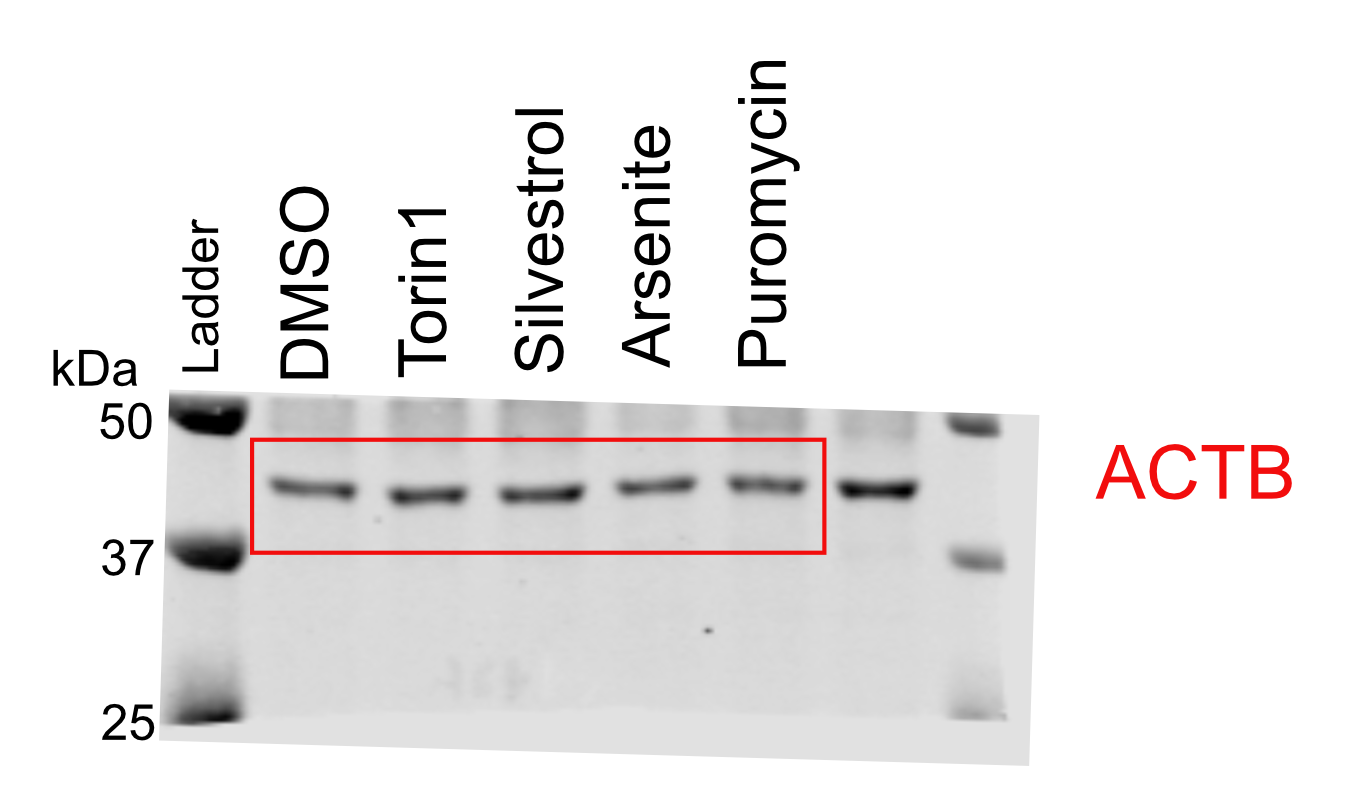

Supplement: Supplementary file 5 — Source data Fig. 3 [file 44318_2024_294_MOESM5_ESM.zip › Figure_3/3C/western_ACTB.tiff]

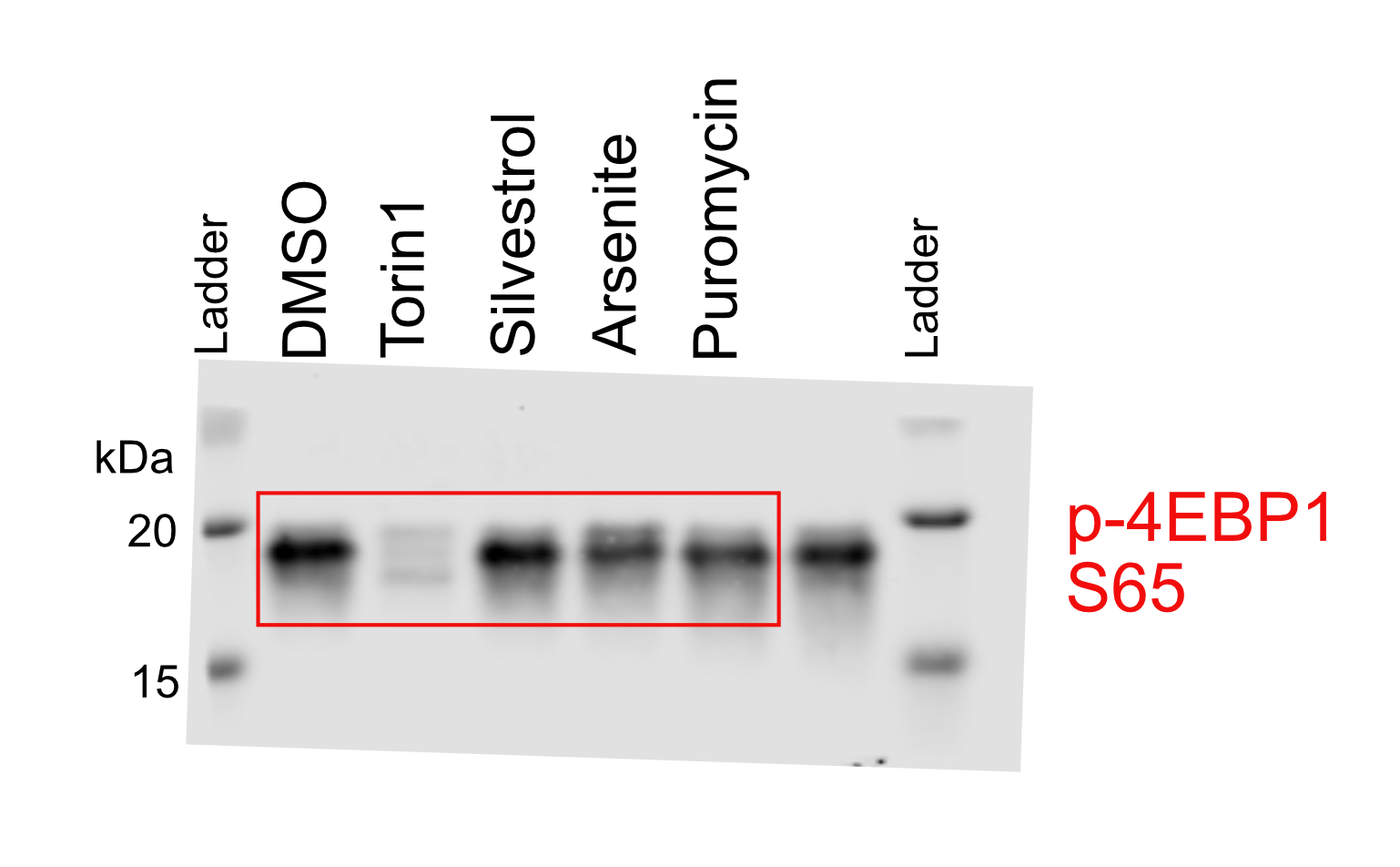

Supplement: Supplementary file 5 — Source data Fig. 3 [file 44318_2024_294_MOESM5_ESM.zip › Figure_3/3C/western_p-4EBP1-s65.tiff]

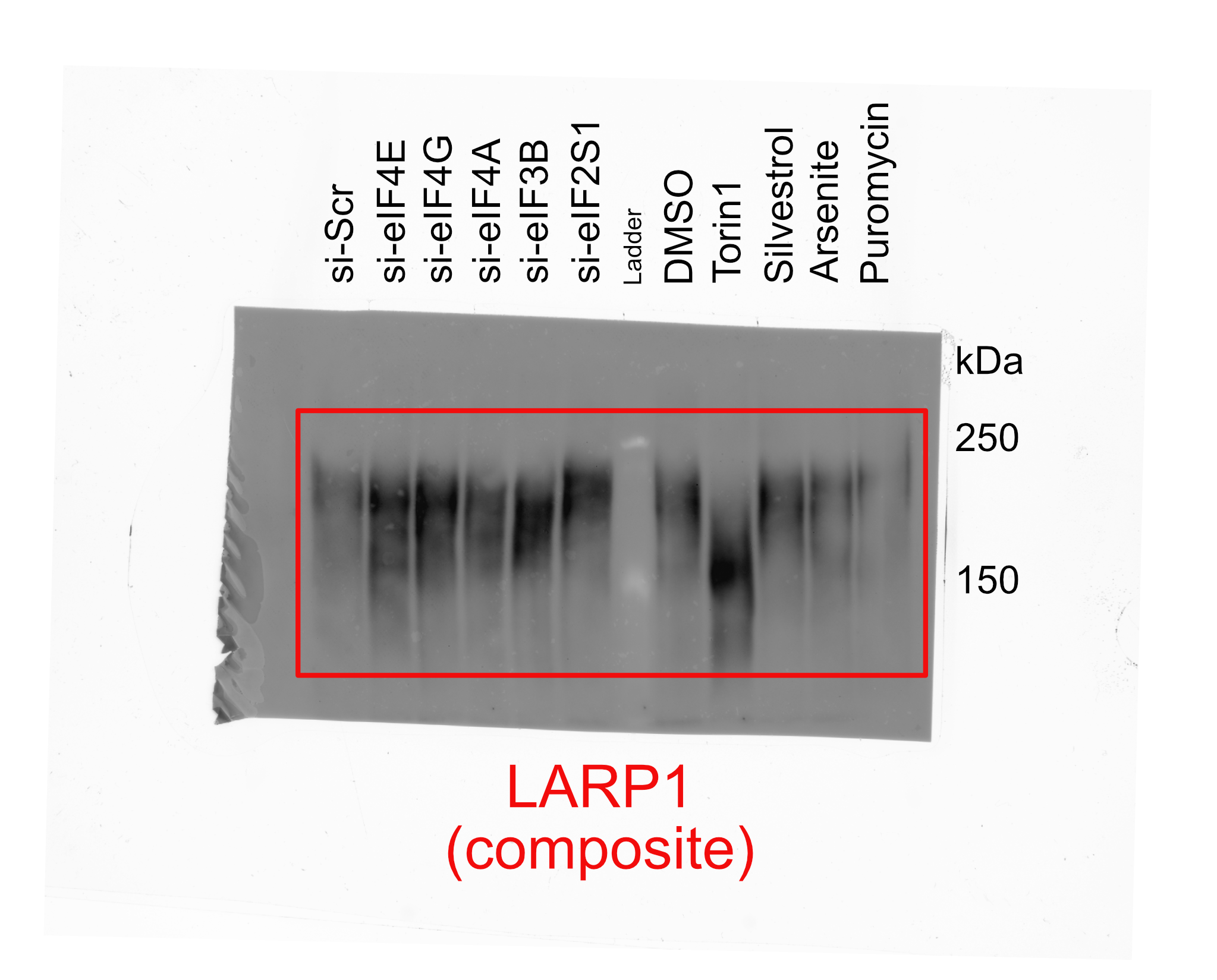

Supplement: Supplementary file 5 — Source data Fig. 3 [file 44318_2024_294_MOESM5_ESM.zip › Figure_3/3D/phostag_composite.tiff]

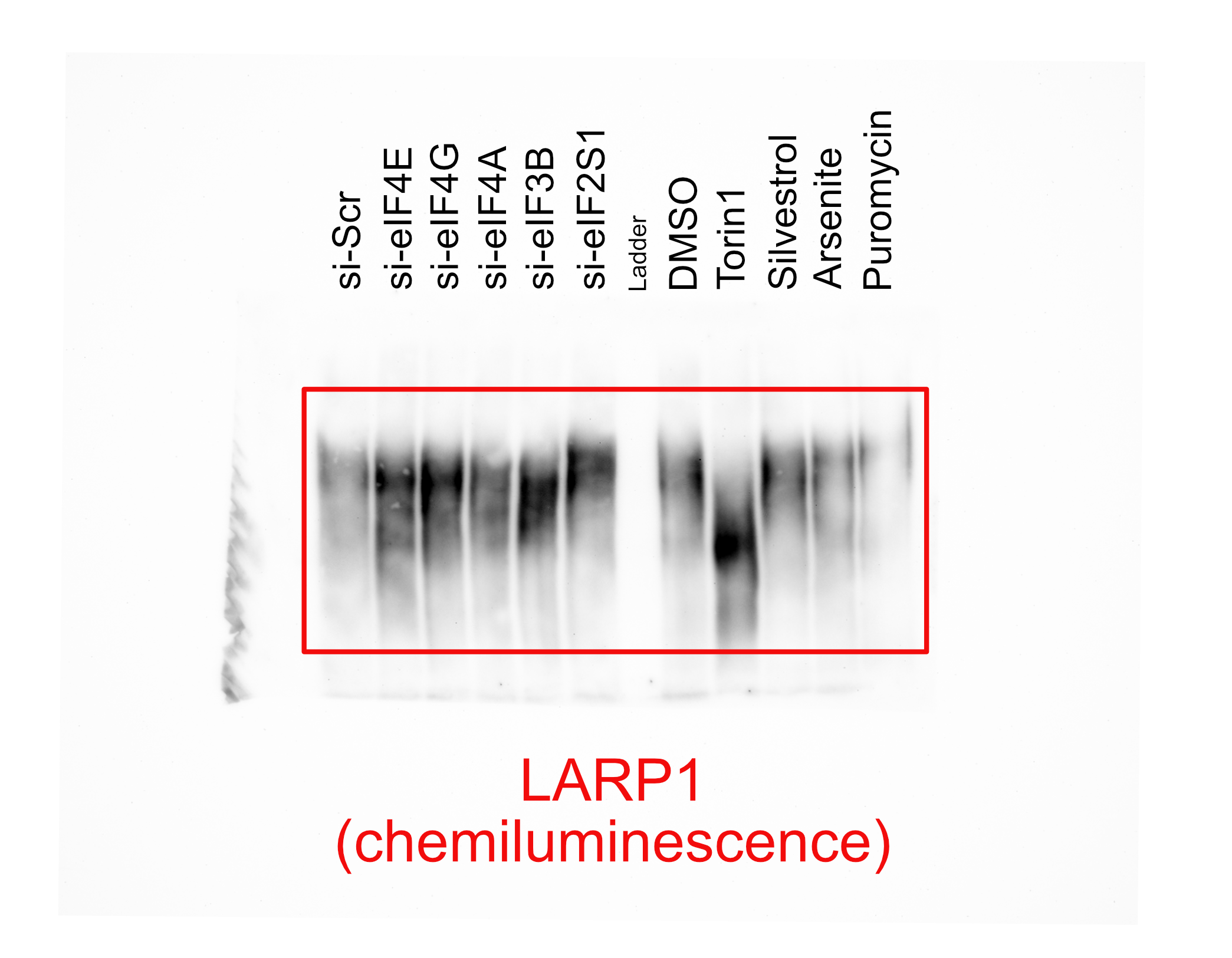

Supplement: Supplementary file 5 — Source data Fig. 3 [file 44318_2024_294_MOESM5_ESM.zip › Figure_3/3D/phostag_chemiluminescence.tiff]

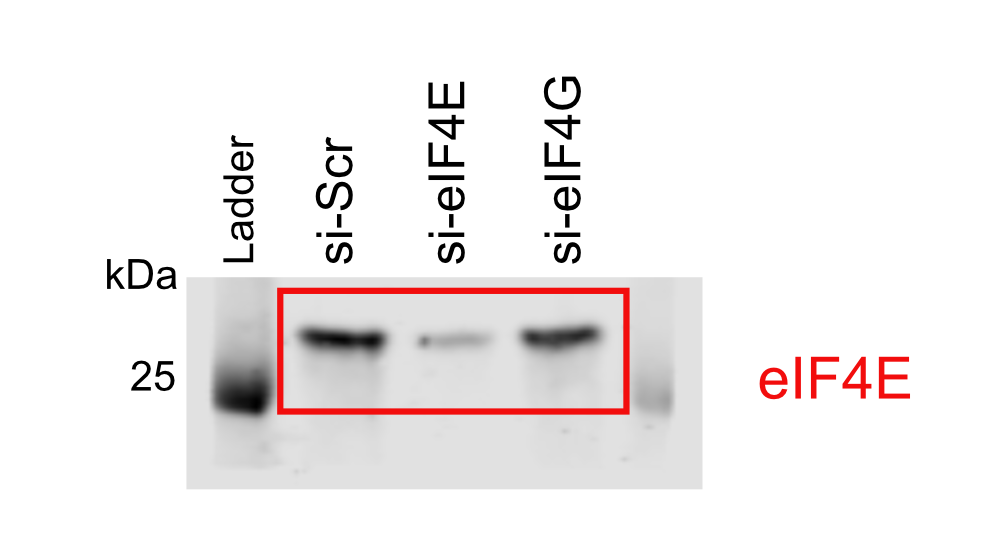

Supplement: Supplementary file 5 — Source data Fig. 3 [file 44318_2024_294_MOESM5_ESM.zip › Figure_3/3A/western_eIF4E.tiff]

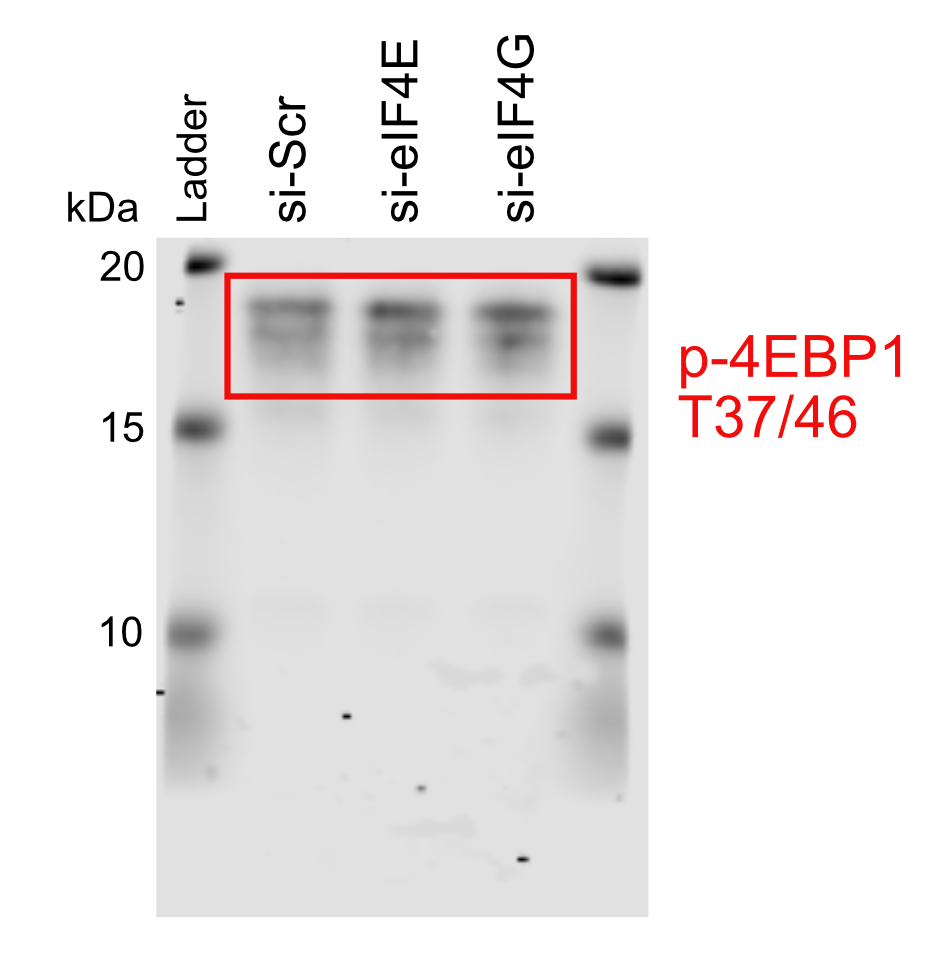

Supplement: Supplementary file 5 — Source data Fig. 3 [file 44318_2024_294_MOESM5_ESM.zip › Figure_3/3A/western_p-4EBP1-t37-t46.tiff]

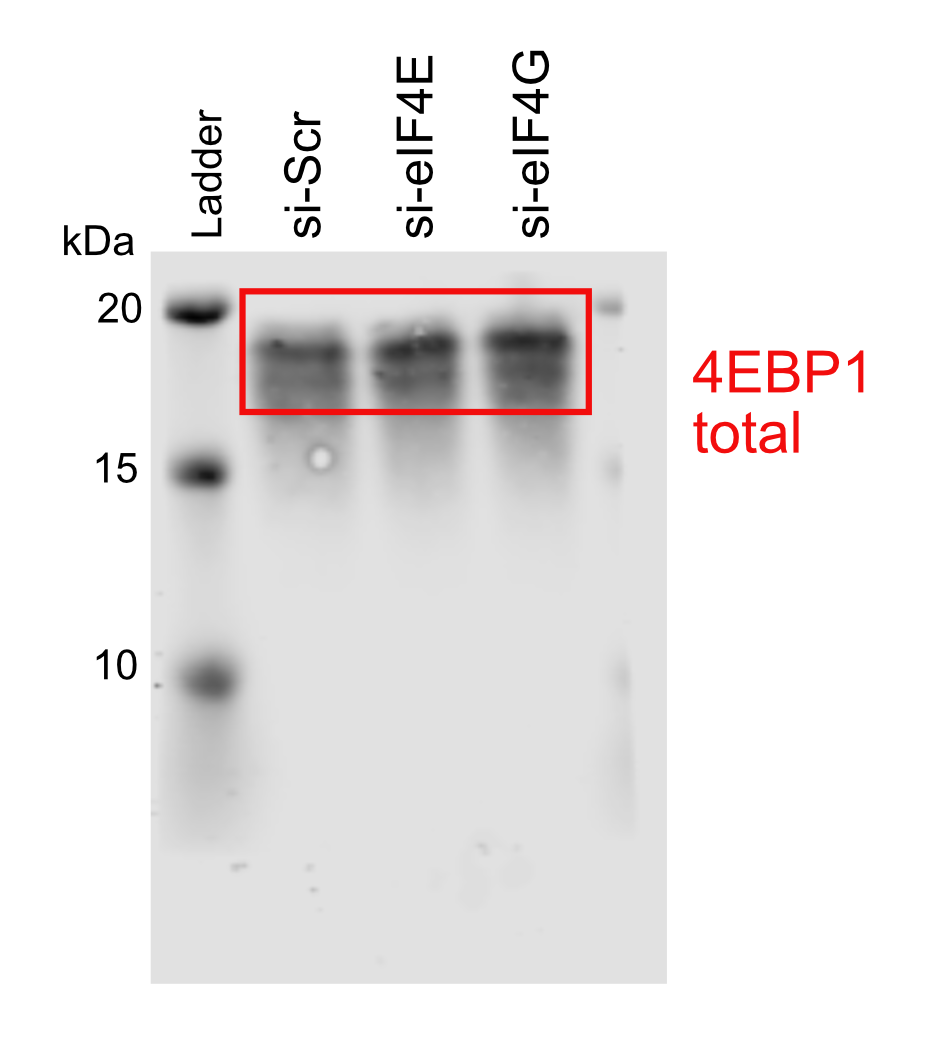

Supplement: Supplementary file 5 — Source data Fig. 3 [file 44318_2024_294_MOESM5_ESM.zip › Figure_3/3A/western_4EBP1-total.tiff]

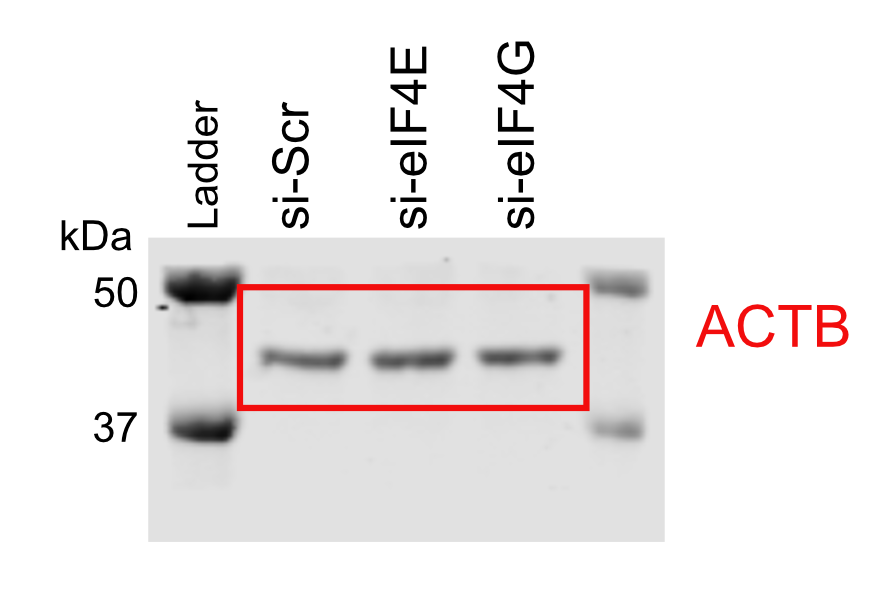

Supplement: Supplementary file 5 — Source data Fig. 3 [file 44318_2024_294_MOESM5_ESM.zip › Figure_3/3A/western_ACTB.tiff]

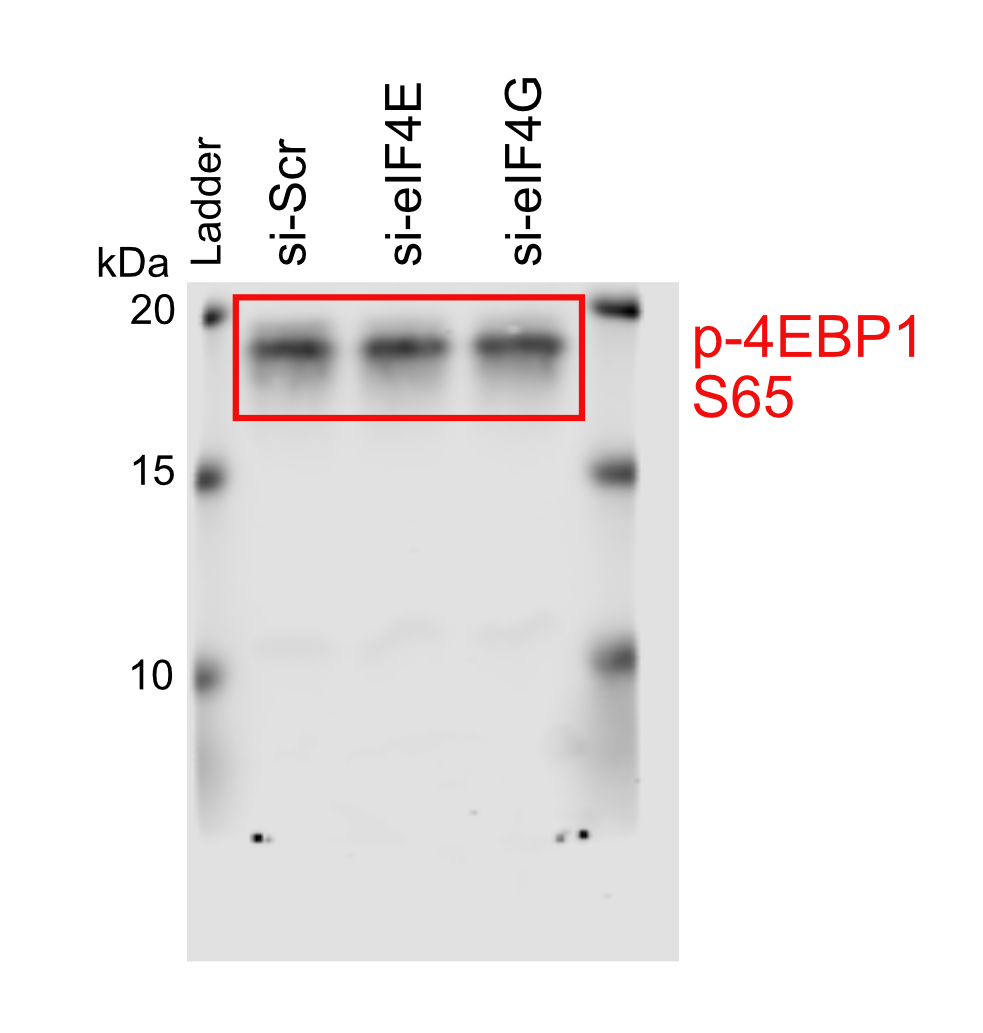

Supplement: Supplementary file 5 — Source data Fig. 3 [file 44318_2024_294_MOESM5_ESM.zip › Figure_3/3A/western_p-4EBP1-s65.tiff]

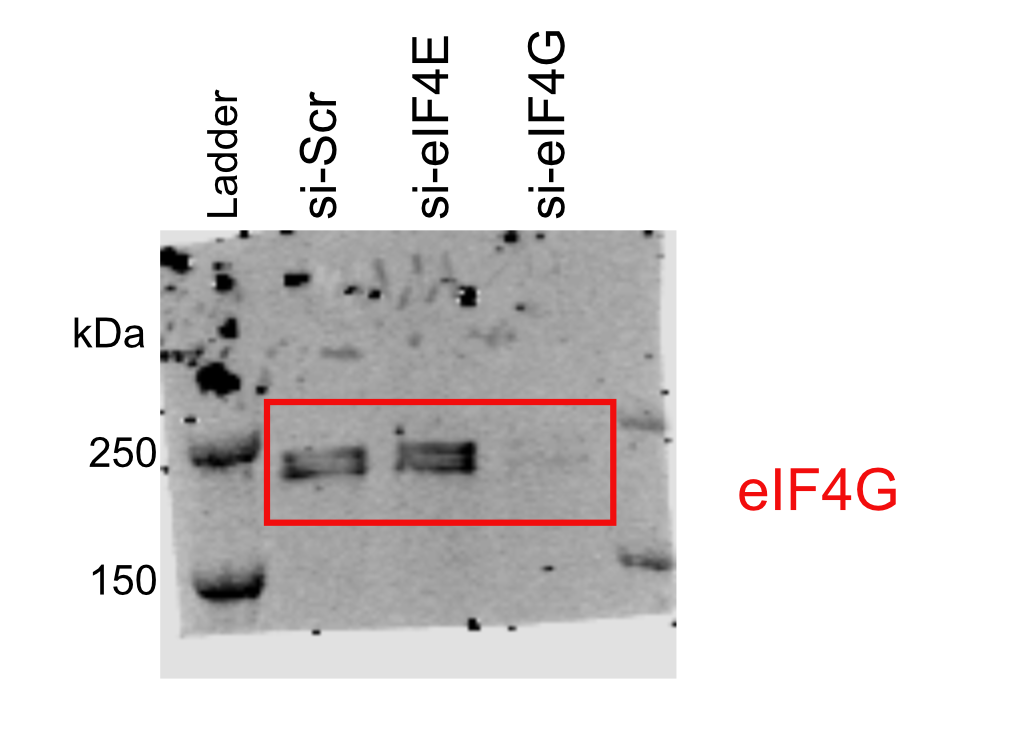

Supplement: Supplementary file 5 — Source data Fig. 3 [file 44318_2024_294_MOESM5_ESM.zip › Figure_3/3A/western_eIF4G.tiff]

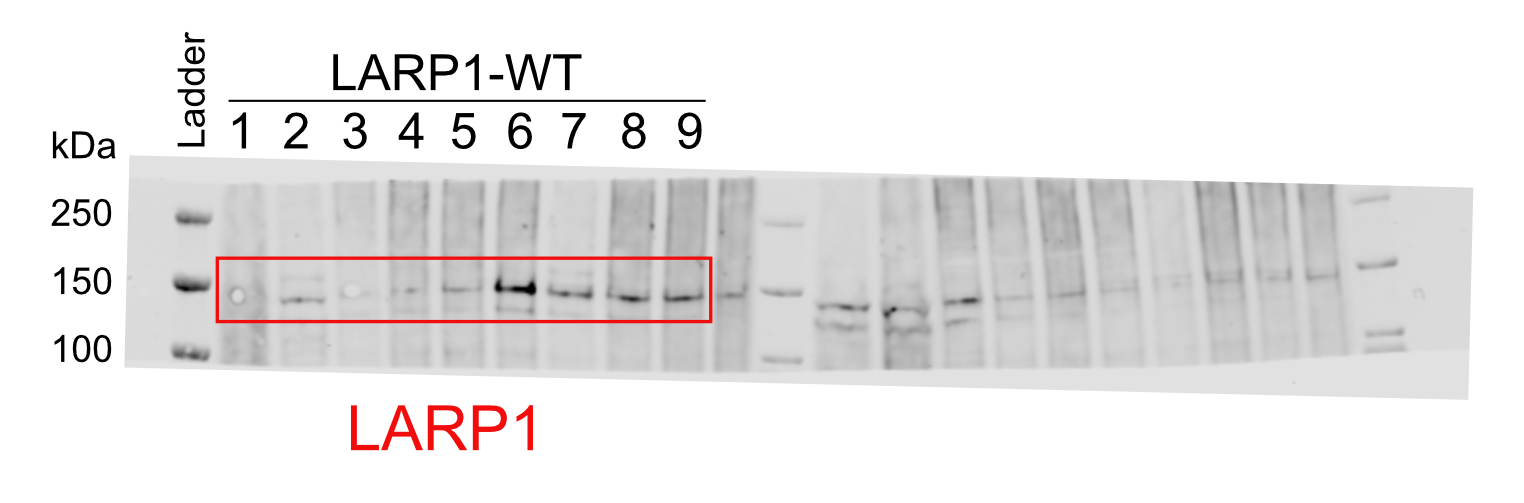

Supplement: Supplementary file 6 — Source data Fig. 5 [file 44318_2024_294_MOESM6_ESM.zip › Figure_5/5C/western_LARP1-WT.tiff]

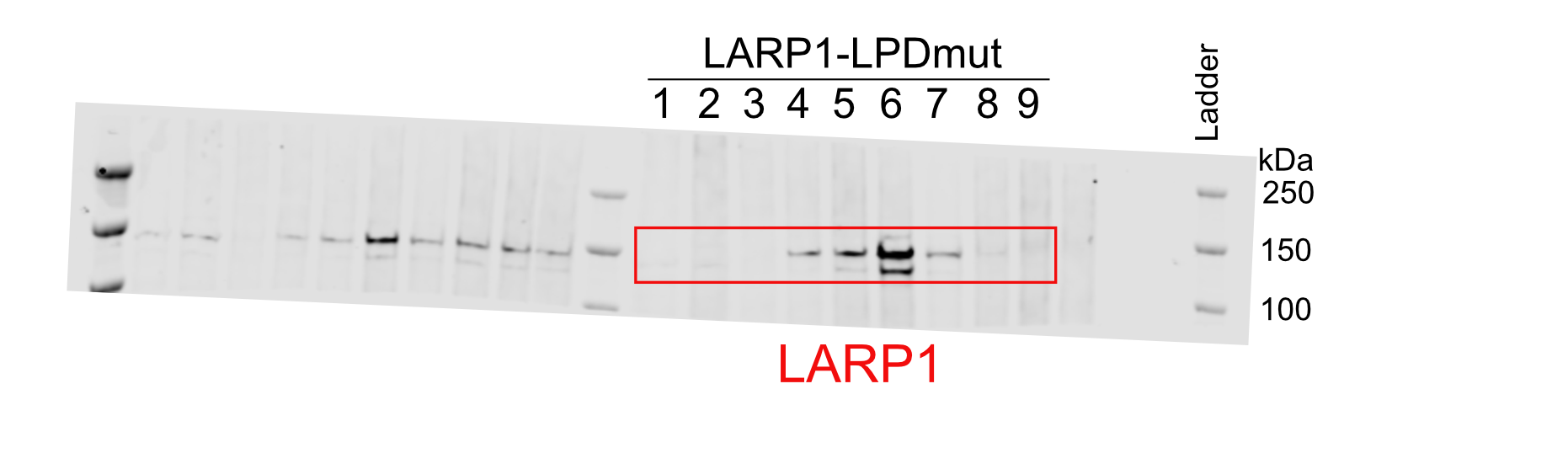

Supplement: Supplementary file 6 — Source data Fig. 5 [file 44318_2024_294_MOESM6_ESM.zip › Figure_5/5C/western_LARP1-LPDmut.tiff]

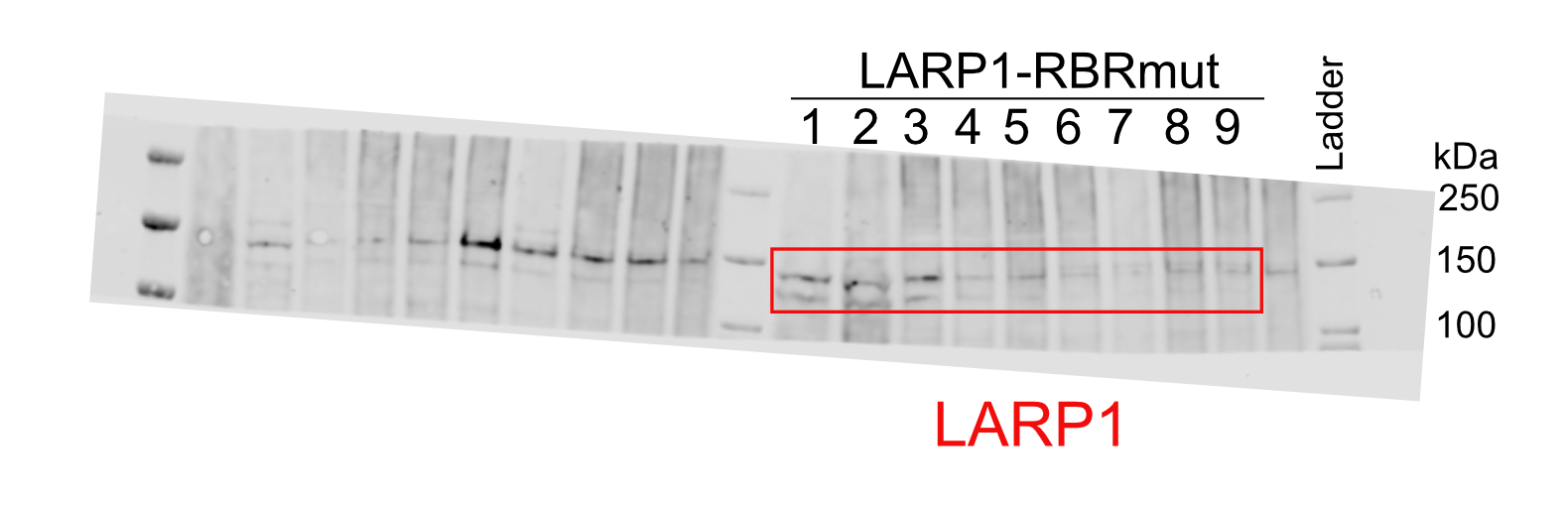

Supplement: Supplementary file 6 — Source data Fig. 5 [file 44318_2024_294_MOESM6_ESM.zip › Figure_5/5C/western_LARP1-RBRmut.tiff]

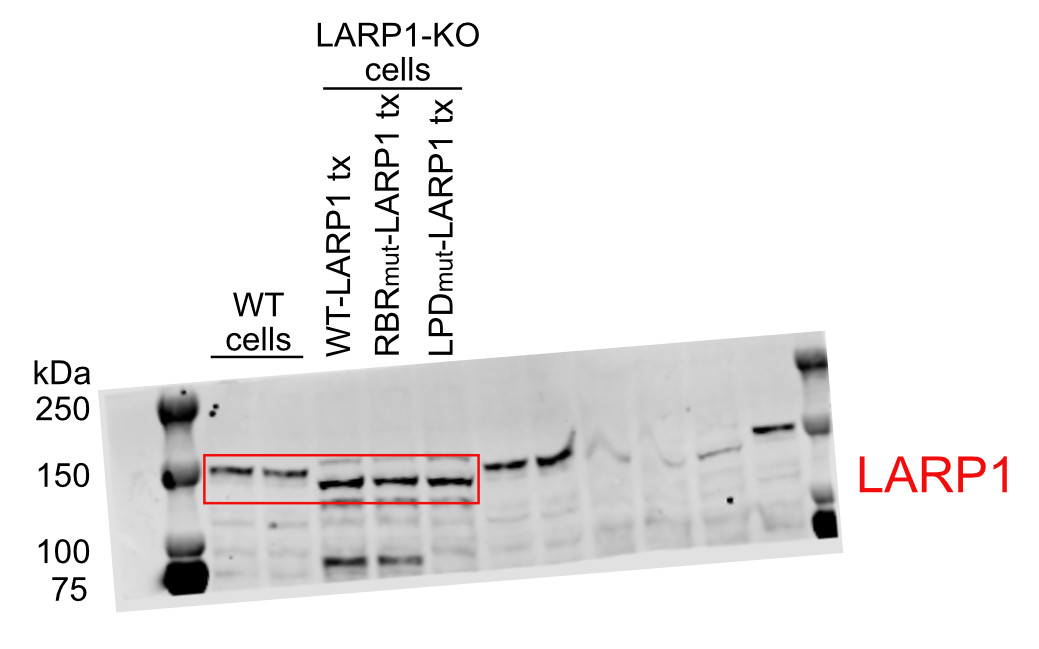

Supplement: Supplementary file 6 — Source data Fig. 5 [file 44318_2024_294_MOESM6_ESM.zip › Figure_5/5B/western_LARP1.tiff]

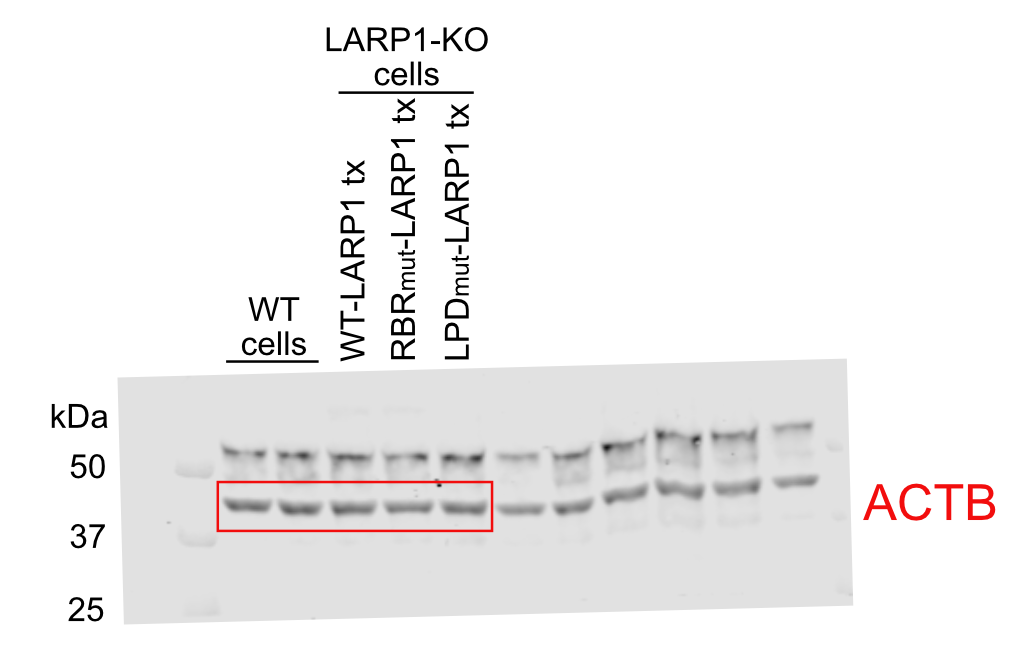

Supplement: Supplementary file 6 — Source data Fig. 5 [file 44318_2024_294_MOESM6_ESM.zip › Figure_5/5B/western_ACTB.tiff]
